# Duplication of specialized *cis*-acting elements SLI and SLIII in the viral RNA 3′UTR enables mosquito-borne orthoflavivirus adaptation to avian hosts

**DOI:** 10.1093/nar/gkag791

**Published:** 2026-08-11

**Authors:** Li Mao, Yu He, Tao Wang, Zhen Wu, Andres Merits, Mingshu Wang, Renyong Jia, Dekang Zhu, Mafeng Liu, Xinxin Zhao, Qiao Yang, Ying Wu, Shaqiu Zhang, Juan Huang, Xumin Ou, Di Sun, Bin Tian, Anchun Cheng, Shun Chen

**Affiliations:** Institute of Veterinary Medicine and Immunology, Sichuan Agricultural University, Chengdu, Sichuan 611130, China; Institute of Veterinary Medicine and Immunology, Sichuan Agricultural University, Chengdu, Sichuan 611130, China; Research Center of Avian Disease, College of Veterinary Medicine, Sichuan Agricultural University, Chengdu, Sichuan 611130, China; Agricultural Animal Diseases and Veterinary Public Health Key Laboratory of Sichuan Province, Sichuan Agricultural University, Chengdu, Sichuan 611130, China; Key Laboratory of Agricultural Bioinformatics, Ministry of Education, Sichuan Agricultural University, Chengdu, Sichuan 611130, China; Engineering Research Center of Southwest Animal Disease Prevention and Control Technology for Ministry of Education, Sichuan Agricultural University, Chengdu, Sichuan 611130, China; Institute of Veterinary Medicine and Immunology, Sichuan Agricultural University, Chengdu, Sichuan 611130, China; Research Center of Avian Disease, College of Veterinary Medicine, Sichuan Agricultural University, Chengdu, Sichuan 611130, China; Agricultural Animal Diseases and Veterinary Public Health Key Laboratory of Sichuan Province, Sichuan Agricultural University, Chengdu, Sichuan 611130, China; Engineering Research Center of Southwest Animal Disease Prevention and Control Technology for Ministry of Education, Sichuan Agricultural University, Chengdu, Sichuan 611130, China; Institute of Veterinary Medicine and Immunology, Sichuan Agricultural University, Chengdu, Sichuan 611130, China; Research Center of Avian Disease, College of Veterinary Medicine, Sichuan Agricultural University, Chengdu, Sichuan 611130, China; Agricultural Animal Diseases and Veterinary Public Health Key Laboratory of Sichuan Province, Sichuan Agricultural University, Chengdu, Sichuan 611130, China; Key Laboratory of Agricultural Bioinformatics, Ministry of Education, Sichuan Agricultural University, Chengdu, Sichuan 611130, China; Engineering Research Center of Southwest Animal Disease Prevention and Control Technology for Ministry of Education, Sichuan Agricultural University, Chengdu, Sichuan 611130, China; Institute of Bioengineering, University of Tartu, Tartu, Estonia; Institute of Veterinary Medicine and Immunology, Sichuan Agricultural University, Chengdu, Sichuan 611130, China; Research Center of Avian Disease, College of Veterinary Medicine, Sichuan Agricultural University, Chengdu, Sichuan 611130, China; Agricultural Animal Diseases and Veterinary Public Health Key Laboratory of Sichuan Province, Sichuan Agricultural University, Chengdu, Sichuan 611130, China; Engineering Research Center of Southwest Animal Disease Prevention and Control Technology for Ministry of Education, Sichuan Agricultural University, Chengdu, Sichuan 611130, China; Institute of Veterinary Medicine and Immunology, Sichuan Agricultural University, Chengdu, Sichuan 611130, China; Research Center of Avian Disease, College of Veterinary Medicine, Sichuan Agricultural University, Chengdu, Sichuan 611130, China; Agricultural Animal Diseases and Veterinary Public Health Key Laboratory of Sichuan Province, Sichuan Agricultural University, Chengdu, Sichuan 611130, China; Engineering Research Center of Southwest Animal Disease Prevention and Control Technology for Ministry of Education, Sichuan Agricultural University, Chengdu, Sichuan 611130, China; Institute of Veterinary Medicine and Immunology, Sichuan Agricultural University, Chengdu, Sichuan 611130, China; Research Center of Avian Disease, College of Veterinary Medicine, Sichuan Agricultural University, Chengdu, Sichuan 611130, China; Agricultural Animal Diseases and Veterinary Public Health Key Laboratory of Sichuan Province, Sichuan Agricultural University, Chengdu, Sichuan 611130, China; Engineering Research Center of Southwest Animal Disease Prevention and Control Technology for Ministry of Education, Sichuan Agricultural University, Chengdu, Sichuan 611130, China; Institute of Veterinary Medicine and Immunology, Sichuan Agricultural University, Chengdu, Sichuan 611130, China; Research Center of Avian Disease, College of Veterinary Medicine, Sichuan Agricultural University, Chengdu, Sichuan 611130, China; Agricultural Animal Diseases and Veterinary Public Health Key Laboratory of Sichuan Province, Sichuan Agricultural University, Chengdu, Sichuan 611130, China; Engineering Research Center of Southwest Animal Disease Prevention and Control Technology for Ministry of Education, Sichuan Agricultural University, Chengdu, Sichuan 611130, China; Institute of Veterinary Medicine and Immunology, Sichuan Agricultural University, Chengdu, Sichuan 611130, China; Research Center of Avian Disease, College of Veterinary Medicine, Sichuan Agricultural University, Chengdu, Sichuan 611130, China; Agricultural Animal Diseases and Veterinary Public Health Key Laboratory of Sichuan Province, Sichuan Agricultural University, Chengdu, Sichuan 611130, China; Engineering Research Center of Southwest Animal Disease Prevention and Control Technology for Ministry of Education, Sichuan Agricultural University, Chengdu, Sichuan 611130, China; Institute of Veterinary Medicine and Immunology, Sichuan Agricultural University, Chengdu, Sichuan 611130, China; Research Center of Avian Disease, College of Veterinary Medicine, Sichuan Agricultural University, Chengdu, Sichuan 611130, China; Agricultural Animal Diseases and Veterinary Public Health Key Laboratory of Sichuan Province, Sichuan Agricultural University, Chengdu, Sichuan 611130, China; Engineering Research Center of Southwest Animal Disease Prevention and Control Technology for Ministry of Education, Sichuan Agricultural University, Chengdu, Sichuan 611130, China; Institute of Veterinary Medicine and Immunology, Sichuan Agricultural University, Chengdu, Sichuan 611130, China; Research Center of Avian Disease, College of Veterinary Medicine, Sichuan Agricultural University, Chengdu, Sichuan 611130, China; Agricultural Animal Diseases and Veterinary Public Health Key Laboratory of Sichuan Province, Sichuan Agricultural University, Chengdu, Sichuan 611130, China; Engineering Research Center of Southwest Animal Disease Prevention and Control Technology for Ministry of Education, Sichuan Agricultural University, Chengdu, Sichuan 611130, China; Institute of Veterinary Medicine and Immunology, Sichuan Agricultural University, Chengdu, Sichuan 611130, China; Research Center of Avian Disease, College of Veterinary Medicine, Sichuan Agricultural University, Chengdu, Sichuan 611130, China; Agricultural Animal Diseases and Veterinary Public Health Key Laboratory of Sichuan Province, Sichuan Agricultural University, Chengdu, Sichuan 611130, China; Engineering Research Center of Southwest Animal Disease Prevention and Control Technology for Ministry of Education, Sichuan Agricultural University, Chengdu, Sichuan 611130, China; Institute of Veterinary Medicine and Immunology, Sichuan Agricultural University, Chengdu, Sichuan 611130, China; Research Center of Avian Disease, College of Veterinary Medicine, Sichuan Agricultural University, Chengdu, Sichuan 611130, China; Agricultural Animal Diseases and Veterinary Public Health Key Laboratory of Sichuan Province, Sichuan Agricultural University, Chengdu, Sichuan 611130, China; Engineering Research Center of Southwest Animal Disease Prevention and Control Technology for Ministry of Education, Sichuan Agricultural University, Chengdu, Sichuan 611130, China; Institute of Veterinary Medicine and Immunology, Sichuan Agricultural University, Chengdu, Sichuan 611130, China; Research Center of Avian Disease, College of Veterinary Medicine, Sichuan Agricultural University, Chengdu, Sichuan 611130, China; Agricultural Animal Diseases and Veterinary Public Health Key Laboratory of Sichuan Province, Sichuan Agricultural University, Chengdu, Sichuan 611130, China; Key Laboratory of Agricultural Bioinformatics, Ministry of Education, Sichuan Agricultural University, Chengdu, Sichuan 611130, China; Engineering Research Center of Southwest Animal Disease Prevention and Control Technology for Ministry of Education, Sichuan Agricultural University, Chengdu, Sichuan 611130, China; Institute of Veterinary Medicine and Immunology, Sichuan Agricultural University, Chengdu, Sichuan 611130, China; Research Center of Avian Disease, College of Veterinary Medicine, Sichuan Agricultural University, Chengdu, Sichuan 611130, China; Agricultural Animal Diseases and Veterinary Public Health Key Laboratory of Sichuan Province, Sichuan Agricultural University, Chengdu, Sichuan 611130, China; Engineering Research Center of Southwest Animal Disease Prevention and Control Technology for Ministry of Education, Sichuan Agricultural University, Chengdu, Sichuan 611130, China; Institute of Veterinary Medicine and Immunology, Sichuan Agricultural University, Chengdu, Sichuan 611130, China; Research Center of Avian Disease, College of Veterinary Medicine, Sichuan Agricultural University, Chengdu, Sichuan 611130, China; Agricultural Animal Diseases and Veterinary Public Health Key Laboratory of Sichuan Province, Sichuan Agricultural University, Chengdu, Sichuan 611130, China; Key Laboratory of Agricultural Bioinformatics, Ministry of Education, Sichuan Agricultural University, Chengdu, Sichuan 611130, China; Institute of Veterinary Medicine and Immunology, Sichuan Agricultural University, Chengdu, Sichuan 611130, China; Research Center of Avian Disease, College of Veterinary Medicine, Sichuan Agricultural University, Chengdu, Sichuan 611130, China; Agricultural Animal Diseases and Veterinary Public Health Key Laboratory of Sichuan Province, Sichuan Agricultural University, Chengdu, Sichuan 611130, China; Engineering Research Center of Southwest Animal Disease Prevention and Control Technology for Ministry of Education, Sichuan Agricultural University, Chengdu, Sichuan 611130, China; Institute of Veterinary Immunology and Green Drugs, Veterinary Department in College of Animal Science, State Key Laboratory of Green Pesticide, Guizhou University, Guiyang 550025, China; Institute of Veterinary Medicine and Immunology, Sichuan Agricultural University, Chengdu, Sichuan 611130, China; Research Center of Avian Disease, College of Veterinary Medicine, Sichuan Agricultural University, Chengdu, Sichuan 611130, China; Agricultural Animal Diseases and Veterinary Public Health Key Laboratory of Sichuan Province, Sichuan Agricultural University, Chengdu, Sichuan 611130, China; Key Laboratory of Agricultural Bioinformatics, Ministry of Education, Sichuan Agricultural University, Chengdu, Sichuan 611130, China; Engineering Research Center of Southwest Animal Disease Prevention and Control Technology for Ministry of Education, Sichuan Agricultural University, Chengdu, Sichuan 611130, China

## Abstract

Cross-species transmission of mosquito-borne orthoflaviviruses (MBFVs) relies on multi-host switching and adaptation, yet the underlying mechanisms remain incompletely understood. Domain I of the 3′-untranslated region (UTR) is highly structured and shaped by host-driven selection. Mammalian MBFVs usually have two functional stem–loops (SLs), while avian MBFVs possess two extra SLs (SLI and SLIII), whose functions remain unclear. Using Tembusu virus (TMUV) and Japanese encephalitis virus (JEV), we mutated Domain I and characterized recombinant viruses. TMUV mutants with only one SL showed attenuated replication, with efficiency dependent on SL identity. Duplicating SLII boosted TMUV replication in mammalian and avian cells but not in mosquito cells, indicating that repeated SLs contribute to host-specific proliferation. Deleting SLI and/or SLIII impaired TMUV replication in avian cells but not mammalian cells, and dual deletion significantly reduced virulence in ducklings but had limited impact in mice. Replacement of JEV SLI/SLIII with homologous elements derived from a duck-associated strain improved its proliferation in avian cells. Mechanistically, SLI/SLIII regulate TMUV replication in avian cells by interacting with the host factor CELF2, which specifically facilitates viral replication in avian cells. Collectively, SLI and SLIII are important determinants of avian host proliferation in MBFVs, improving understanding of their multi-host ecology and transmission.

## Graphical Abstract

**Graphical Abstract:**
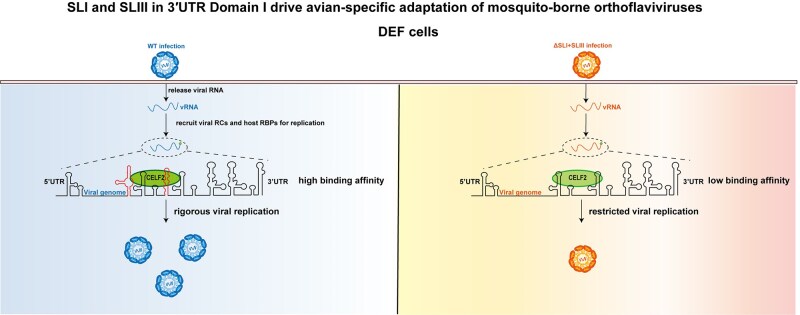

## Introduction

Orthoflaviviruses are globally distributed positive-sense RNA viruses that infect a wide range of vertebrate hosts and are primarily transmitted by arthropod vectors. Based on their ecological and transmission characteristics, they are divided into four groups: mosquito-borne orthoflaviviruses (MBFVs), tick-borne orthoflaviviruses (TBEVs), insect-specific orthoflaviviruses (ISFVs), and no-known vector orthoflaviviruses (NKVFs) [1]. Among these, MBFVs are the most extensively studied because of their major impacts on human and animal health. They are often classified into two groups: primate-related orthoflaviviruses, which are maintained in a human–mosquito–human transmission cycle, and avian-related orthoflaviviruses, in which birds serve as amplification or reservoir hosts while mammals typically function as dead-end hosts. An exception is Japanese encephalitis virus (JEV), for which pigs also act as important amplification hosts [2]. Several MBFVs—including dengue virus (DENV), Zika virus (ZIKV), West Nile virus (WNV), and JEV—are major human pathogens that impose substantial global medical and economic burdens [3, 4]. Birds play a central role in the transmission ecology of many MBFVs and have been implicated in outbreaks of WNV in North America [5], JEV and Tembusu virus (TMUV) in Asia [6, 7], and Usutu virus (USUV) in Europe [8]. Notably, >90 avian species, encompassing both domestic and wild birds, are susceptible to JEV infection and can develop varying levels of viraemia following natural or experimental infection [9, 10], highlighting the critical role of birds in the ecology and geographic spread of MBFVs.

The MBFV genome is ~1 kb in length and contains a single main open reading frame (ORF) flanked by 5′- and 3′-untranslated regions (UTRs). The 3′UTR comprises three domains. Domain I, also known as the variable region (VR), is located downstream of the ORF stop codon and contains a variable number of stem–loop (SL) structures. Domain II is moderately conserved and, in most MBFVs, contains two dumbbell (DB) structures. Domain III is the most conserved region and includes a small hairpin (sHP) and 3′SL structures [11]. Domain I of DENV and ZIKV, which use mammals as amplification hosts, contains two similar SLs designated SLI and SLII [12, 13]. In contrast, MBFVs that use birds as amplification hosts—including those in the JEV subgroup (e.g. JEV, USUV, and WNV) and the Ntaya virus subgroup (e.g. Bagaza virus and TMUV)—possess more complex Domain I structures with four SLs: a pair of duplicated SLs (SLII and SLIV), which exhibit conserved structural blocks analogous to those found in DENV and related viruses, as well as two unique avian orthoflavivirus SLs (SLI and SLIII) that precede and intercalate them [14, 15, 16].

Numerous studies have elucidated the biological significance of conserved, duplicated SL structures within Domain I of MBFVs. These SLs are involved in viral RNA replication [17, 18], cap-independent translation [14, 19], regulation of pathogenicity [15, 20], subgenomic flaviviral RNA (sfRNA) generation [21, 22], and host-specific adaptation. Regarding adaptation, DENV and ZIKV have evolved SL structures with inverted positions but similar functions: deletion of ZIKV SLI or DENV SLII enhanced viral replication in mosquito cells with little effect in mammalian cells [12, 13]. However, deletion of both ZIKV SLI and SLII abolished replication in mammalian cells while maintaining infectivity in mosquito cells [13]. Strikingly, introduction of ZIKV SLI into Donggang virus (DONV, a dual-host ISFV) promoted viral RNA replication in human kidney cells (HEK-293T) [1]. Likewise, replacing the SLI of mosquito-derived TMUV with the SLI from duck-derived TMUV enhanced viral proliferation in both mammalian and avian cells [23]. Despite these insights, the specific roles of SLI and SLIII in adaptation to avian hosts remain poorly characterized.

In this study, we investigated the role of RNA structural elements within Domain I of the 3′UTR in avian orthoflavivirus host adaptation. We generated a series of recombinant TMUV and JEV genomes carrying deletions, substitutions, or exchanges of SL structures in Domain I and evaluated their infectivity, replication kinetics in cells from different hosts, and *in vivo* virulence. We found that SLI and SLIII of TMUV and JEV are important determinants of efficient proliferation in avian cells, but are non-essential in mammalian and mosquito cells. RNA pulldown coupled with mass spectrometry (MS) identified CUGBP Elav-like family member 2 (CELF2) as an SLI/SLIII-associated host factor that promotes TMUV proliferation specifically in avian cells. Together, these findings demonstrate the critical importance of these SL structures in avian-adapted MBFVs and deepen our understanding of the *cis-*acting RNA elements that support their multi-host lifestyle.

## Materials and methods

### Ethics statement

All animal experimental procedures were approved by the Institutional Animal Care and Use Committee of Sichuan Agricultural University, China (Protocol Permit Number: 20 260 030).

### Cells and viruses

Primary duck embryonic fibroblast (DEF) cells, isolated from 9-day-old duck embryos, human embryonic kidney cells (HEK-293T, ATCC CRL-3216), and baby hamster kidney (BHK-21, ATCC CCL-10) cells were cultured in Dulbecco’s modified Eagle’s medium (DMEM; Gibco, Shanghai, China) supplemented with 10% foetal bovine serum (FBS; KEL, Shanghai, China) and 0.001% ciprofloxacin. Cultures were maintained at 37°C in 5% CO_2_. *Aedes albopictus*-derived C6/36 cells were maintained in RPMI 1640 medium (Gibco, Shanghai, China) supplemented with 10% FBS and 0.001% ciprofloxacin at 28°C without additional CO_2_.

JEV strain duck/2022-SD-1 (SD-1; GenBank ID: OR711406.1) was gifted by Chenxi Li (Yangzhou University, China). TMUV strain CQW1 (GenBank ID: KM233707.1), JEV strain SA14 (GenBank ID: KU323483.1), and all related mutant viruses were rescued from infectious cDNA (icDNA) clones. Virus stocks were amplified in BHK-21 cells, aliquoted, and stored at −80°C.

### Construction of plasmids harbouring cDNAs of mutant TMUV replicons

To construct TMUV 3′UTR mutant replicons, a plasmid harbouring cDNA of mC-Replicon-SecNLuc [24], designated here as the wild type (WT), was modified by replacing the SbfI–NotI fragment with fragments containing the intended mutations. These fragments corresponded to mutant 3′UTRs preceded by partial NS5-encoding sequences and were obtained by amplification of overlapping PCR fragments using oligonucleotide primers listed in Supplementary Table S1. Sequence data for DENV SLI (ID: AF038403) and ZIKV Domain I (ID: KU365778) were obtained from the NCBI database.

### Replicon assay and nanoluciferase activity detection

BHK-21 cells were seeded in 48-well plates and transfected with 0.2 µg of WT or mutant replicon plasmids using TransIntro EL Transfection Reagent (TransGen Biotech, Beijing, China) according to the manufacturer’s protocol. At 12, 24, 48, and 72 hours post-transfection (hpt), cells were washed once with phosphate-buffered saline (PBS), lysed in 50 µl of Glo Lysis Buffer (Promega, USA) at room temperature for 5 min, and stored at −20°C until analysis.

Nanoluciferase (NLuc) activity was measured using the Nano-Glo Luciferase Assay System (Promega). Briefly, 10 µl of cell lysate and 50 µl of Nano-Glo reagent were added to white 96-well tissue culture plates, mixed, and luminescence was measured using a GloMax Navigator System (Promega). Fluorescence values were normalized to 12 hpt.

### Construction and rescue of recombinant viruses

An icDNA clone of the TMUV CQW1 strain, pACNR-CQW1-Intron [25], was used as the backbone for construction of all mutant TMUV icDNAs, following the same strategy as described above for replicon construction. Mutant cDNA fragments (primers listed in Supplementary Table S1) were used to replace the corresponding WT sequences employing the SbfI and NruI restriction enzymes (NEB, Beijing, China). An icDNA clone of the JEV SA14 strain, pACNR-FL-SA14-intron*2, was generated in our laboratory and used as the backbone for construction of JEV recombinants carrying 3′UTR swaps. Mutations were introduced by fusion PCR (primers listed in Supplementary Table S1), and the WT fragments were replaced with the corresponding mutant fragments using XbaI and SbfI restriction enzymes.

To rescue recombinant viruses, 1.6 or 0.8 µg of each icDNA plasmid was transfected into 70–90% confluent BHK-21 cells in 12-well plates using TransIntro EL Transfection Reagent. At 48–96 hpt, supernatants (F0 virus) were harvested and used to infect fresh BHK-21 cells. Supernatants (F1 virus) were collected upon the appearance of a clear cytopathic effect (CPE) and used in subsequent experiments. Viral replication was confirmed by indirect immunofluorescence assay (IFA) in BHK-21 cells [23].

### Viral titration and growth curve analysis

Viral titres were determined by tissue culture infectious dose 50 (TCID_50_) assays, calculated using the Kärber method. Plaque assays were performed in BHK-21 cells as described previously [26].

For *in vitro* replication assays, BHK-21, DEF, and C6/36 cells were seeded in 24-well plates. At 70–90% confluence, cells were washed twice with PBS and infected with WT or recombinant viruses at 200 TCID_50_/well for 1.5 h at 37°C (BHK-21, DEF) or 28°C (C6/36). After washing, cells were maintained in DMEM or RPMI 1640 medium containing 2% FBS and 0.001% ciprofloxacin. Supernatants were harvested at the indicated times and titrated.

### Serial passage and 3′UTR sequence analysis

TMUV WT virus was rescued in BHK-21 cells serially passaged in BHK-21 or C6/36 cells to assess the stability of 3′UTR Domain I under host-restricted replication conditions. For passage in BHK-21 cells, virus was passaged for 10 consecutive rounds; for passage in C6/36 cells, virus was passaged for five consecutive rounds. Successive infections in the same cell line were performed at 200 TCID_50_. At each passage, culture supernatant from infected cells was harvested, subjected to titration, and used to infect fresh cells of the same type. Viral RNA was extracted from the final passage supernatants using the TIANamp Virus RNA Kit (Tiangen, Beijing, China), and used for reverse transcription–PCRs (RT–PCRs). The sequencing primers 9F (5′-GTGGTTCCATGTCGAGACCAGGATG-3′) and p9R (5′-AGATCCTGTGTTCTACCACCACCAG-3′) were targeted to amplify the TMUV partial NS5 coding region and the whole 3′UTR. The PCR products were subjected to next-generation sequencing to analyse sequence variation within the 3′UTR by the Sangon company (Shanghai, China). Single-nucleotide polymorphisms (SNPs) identified in the 3′UTR were mapped to individual SL elements, and their effects on RNA secondary structure were evaluated by structure prediction.

### Animal experiments

#### Ducklings

Specific pathogen-free (SPF) ducklings (Shandong Haotai Laboratory Animal Breeding Co., Ltd) were housed in negative-pressure isolators immediately after hatching. Five-day-old ducklings were intramuscularly inoculated with 200 µl of WT TMUV, ΔSLI, ΔSLIII, or ΔSLI + SLIII virus at 10^4.8^ TCID_50_ (*n* = 8 per group for survival analysis and *n* = 3 per group for viraemia analysis). Mock-infected controls received DMEM. Animals were monitored daily for survival, clinical signs, and body weight changes. Humane endpoints were defined as body weight loss exceeding 20%, failure to gain weight for three consecutive days, or complete leg paralysis, at which point ducklings were euthanized to minimize suffering. Blood samples were collected at 1 day post-infection (dpi), and viraemia was determined by titration on BHK-21 cells.

#### Mice

Two-week-old Kunming mice (Sichuan Dashuo Laboratory Animal Breeding Co., Ltd) were divided into experimental and mock groups (*n* = 10 per group for the survival experiment and *n* = 3 per group for brain titre determination; both male and female mice were used). Mice were intracranially injected with 30 µl of WT TMUV, ΔSLI, ΔSLIII, or ΔSLI + SLIII virus at 10^4.7^ TCID_50_. Mock-infected controls received DMEM. Body weight, clinical signs, and mortality were monitored daily. Mice were humanely euthanized if body weight loss exceeded 20% or if complete hind-leg paralysis occurred. For detection of virus in the brain, mice were sacrificed at 5 dpi, and brain viral loads were measured by titration in BHK-21 cells.

### Sequence alignment and RNA secondary structure prediction

Pairwise alignment of JEV SA14 and JEV SD-1 SLI/SLIII nucleotide sequences was performed in Geneious software using the ClustalW algorithm. RNA secondary structures of dual-host insect-specific flavivirus (dISFs), avian-related MBFVs, and non-avian MBFVs were predicted using RNAfold (http://rna.tbi.univie.ac.at), UNAFold (http://www.unafold.org/), and VARNA software, then manually refined. Phylogenetic trees were generated in MEGA7 using the Neighbor–Joining (NJ) method. Sequence information was obtained from NCBI for DONV (MZ357894), DENV (AF038403), ZIKV (KU365778), TMUV (KM233707.1), Bagaza virus (BAGV) (PP236852.1), Israel turkey meningoencephalomyelitis virus (ITV) (KC734549.1), WNV (DQ211652.1), Kunjin virus (KUNV) (AY274504), USUV (AY453411.1), and JEV (OR711406.1). Binjari virus (BinJV) sequences were obtained from reference [2].

### RNA extraction and quantitative reverse transcription–PCR

Total RNA was extracted using RNA iso Plus reagent (Takara, Dalian, China) according to the manufacturer’s instructions. RNA concentration and purity were assessed by spectrophotometry (*A*_260_/*A*_280_ ratio). Then 0.5 μg of total RNA was reverse transcribed using the All-in-One First-Strand Synthesis MasterMix (with dsDNase) (YuGong Biotech, Jiangsu, China). To measure viral RNA or the mRNA of interferon-β (IFN-β), quantitative PCR (qPCR) assays were performed using a CFX Connect Real-Time PCR Detection System (Bio-Rad) following the manufacturer’s protocols. Reactions were performed in 96-well plates using 0.4 μl of the reverse transcription reaction mixture as the template, 5 μl of 2× Taq SYBR Green qPCR Premix (Innovagene, Changsha, China), 300 nM of each primer, and RNase-free water to a total volume of 10 μl. Relative IFN-β expression was calculated using the 2^−ΔΔCT^ method with β-actin (DEF cells) or glyceraldehyde phosphate dehydrogenase (GAPDH; HEK-293T cells) as the internal control. All experiments were performed with three biological replicates. All related primers for RT–qPCR are listed in Supplementary Table S1.

### Construction of reporters, *in vitro* transcription, RNA transfection, and *in vitro* translation assay

The DNA fragments corresponding to the reporter RNAs (TMUV 3′UTR WT-Fluc and TMUV 3′UTRΔSLI + SLIII-Fluc), as well as the control reporter (huGL-RLuc), were cloned into the pMD19-T vector (Takara, Japan). A bacteriophage T7 RNA polymerase promoter was inserted upstream of each reporter sequence, and an XhoI restriction site was introduced downstream of the 3′ end. The plasmids containing the reporters were linearized using the XhoI endonuclease (New England Biolabs, USA) and transcribed *in vitro* using a mMESSAGE mMACHINE T7 Transcription Kit (Thermo Fisher Scientific, USA).

BHK-21 and DEF cells were seeded into 48-well plates and co-transfected with 0.25 μg of RNA transcripts of huGL-Rluc together with WT-Fluc or ΔSLI + SLIII-Fluc at a 1:19 ratio. At 6 hpt, the cells were harvested and lysed, after which the activities of RLuc and Fluc were detected using a Dual-Luciferase Reporter Assay System (Vazyme, Nanjing, China).

### TMUV replication detection in DEF cells

We constructed a reporter virus to evaluate TMUV replication in DEF cells, based on a previously described strategy with minor modifications [27]. Using the icDNA infectious clone pACNR-CQW1-Intron as the backbone [25], an NLuc-FMDV-2A fusion cassette was inserted immediately downstream of the first 38 amino acids of the viral capsid protein. A CMV promoter and an SV40 polyadenylation signal were incorporated to drive transcription in eukaryotic cells. NLuc activity in DEF cells was measured as described for the replicon assay above.

### Packaging assay

The packaging assay was performed as reported previously [24]. In brief, BHK-21 cells were seeded into 48-well plates and co-transfected with 0.4 μg of replicon plasmid (Replicon-WT or Replicon-3′UTRΔSLI + SLIII) together with the packaging plasmid (pcDNA3.1-C_16_prME) at a 1:3 ratio. At 6 hpt, the medium was replaced with fresh maintenance medium. At 72 hpt, the supernatant containing single-round infectious particles (SRIPs) was collected and used to infect fresh BHK-21 cells. At 24 hpi, cells were lysed for the NLuc activity assay as described above.

### Biotinylated RNA pulldown and mass spectrometry analysis

The TMUV minigenome RNAs [5′ UTR-C34-66nt-3′UTR-WT or 5′ UTR-C34-66nt-3′UTR-ΔSLI + SLIII], containing the 5′UTR, the sequence encoding the first 34 amino acidsof the capsid protein, a 66 nt spacer sequence, and the WT or mutant 3′UTR of the TMUV genome, were *in vitro* transcribed from a DNA template harbouring a T7 promoter using the MEGAscript T7 Transcription Kit (Thermo Fisher Scientific, USA) [27, 28]. The RNAs were then labelled using the Pierce™ RNA 3′ End Desthiobiotinylation Kit (Thermo Fisher Scientific, USA) according to the manufacturer’s instructions, and RNA quality was verified by electrophoresis in 1% formaldehyde-denatured agarose gels.

DEF cells grown in 10 mm culture dishes were lysed with Pierce IP Lysis Buffer (Thermo Fisher Scientific, USA) containing 1× protease inhibitor cocktail by incubation at 4°C for 1 h. RNA–protein complex pulldowns were performed using the Pierce™ Magnetic RNA-Protein Pull-Down Kit (Thermo Fisher Scientific, USA) following the manufacturer’s instructions. In brief, the biotin-labelled RNAs were incubated with streptavidin magnetic beads at room temperature for 30 min, and then the DEF total proteins were added to the RNA–beads complex and incubated at 4°C overnight. The mixture was washed three times with wash buffer and the beads containing pulled-down proteins were subjected to MS analysis by the BioMS company (Beijing, China). The interaction between RNA and identified Flag-tagged CELF2 was analysed by RNA pulldown as described above. The pull-down proteins were separated by sodium dodecylsulfate (SDS)–polyacrylamide gel electrophoresis (PAGE), and then subjected to western blotting with anti-Flag antibody (Smartlifesciences, Cat No. SLAB0102).

### CELF2 overexpression and western blot analysis

The sequence encoding Flag-tagged duck CELF2 was cloned into the pCAGGS vector; the primers used to amplify the fragment are listed in Supplementary Table S1. The plasmid was transfected into DEF and BHK-21 cells. At 36 hpt, the cells were infected with 10^4^ TCID_50_ of TMUV WT or ΔSLI + SLIII. At 24 hpi, the supernatants were harvested for viral titration, and the cells were lysed with RIPA buffer (Thermo Fisher Scientific, Shanghai, China) containing 1× protease inhibitor cocktail (MedChemExpress, Shanghai, China). Proteins were separated by SDS–PAGE and transferred to polyvinylidene fluoride (PVDF) membranes (Bio-Rad). The membranes were blocked with 5% non-fat milk powder in PBS at 37°C for 3 h and then incubated overnight at 4°C with mouse anti-Flag (1:3 000) (Smartlifesciences, Cat No. SLAB0102) and mouse anti-GAPDH (1:3000) (Proteintech, Cat No. 60004-1-Ig) primary antibodies. Horseradish peroxidase (HRP)-labelled goat anti-mouse IgG (1:3000) (Bio-Rad, Cat No. 1 706 515) was used as the secondary antibody. Protein bands were visualized using enhanced chemiluminescence (ECL) detection reagent (Bio-Rad), and band intensities were quantified using ImageJ software.

### RNA interference

All small interfering RNAs (siRNAs) used in this study were synthesized by Sangon (Shanghai, China). The sequences of the siRNA guide strands were as follows: 5′-UUCUCCGAACGUGUCACGU-3′ (siNC), 5′-GGAGCUGUCUACCAGAUUA-3′ (siduckCELF2-242), 5′-GAUGUUUGUUGGACAGAUU-3′ (siduckCELF2-175), 5′-GGCACAGAAUGCAAUCAAA-3′ (sihamsterCELF2-478), and 5′-GGAGCUGUCUACCAGAUCA-3′ (sihamsterCELF2-116). DEF and BHK-21 cells were seeded in 24-well plates and transfected with siRNAs using NeoLNP RNA transfection reagent (SCINDY, Cat. No. SDR8006) according to the manufacturer’s instructions. At 36 hpt, the cells were infected with TMUV WT, TMUVΔSLI + SLIII, JEV SA14, or JEV SD-1 at a dose of 10^4^ TCID_50_. The supernatants were collected for viral titration at 36 hpi, and the cell lysates were collected to assess the knock-down efficiency by RT–qPCR and western blotting with anti-CELF2 antibody (MedChemExpress, Cat. No. YA2480).

### Statistical analysis

Data were analysed using GraphPad Prism 8.0 (La Jolla, CA, USA). Viral titres are presented as means ± standard deviations (SDs). Differences in NLuc activity and viral growth curves were analysed by two-way analysis of variance (ANOVA); CELF2 knockdown efficiency, viral genome copy number, and IFN-β mRNA expression were analysed by one-way ANOVA; and plaque size differences were assessed by Student’s *t*-test or one-way ANOVA. Survival curves of duck embryos, SPF ducklings, and mice were analysed using the log-rank (Mantel–Cox) test. Body weight change data were analysed using multiple *t*-tests with the Holm–Šidák method. In all cases, *P* < 0.05 was considered statistically significant.

## Results

### SL structures within the 3′UTR Domain I are required for TMUV replication

**Figure 1.**
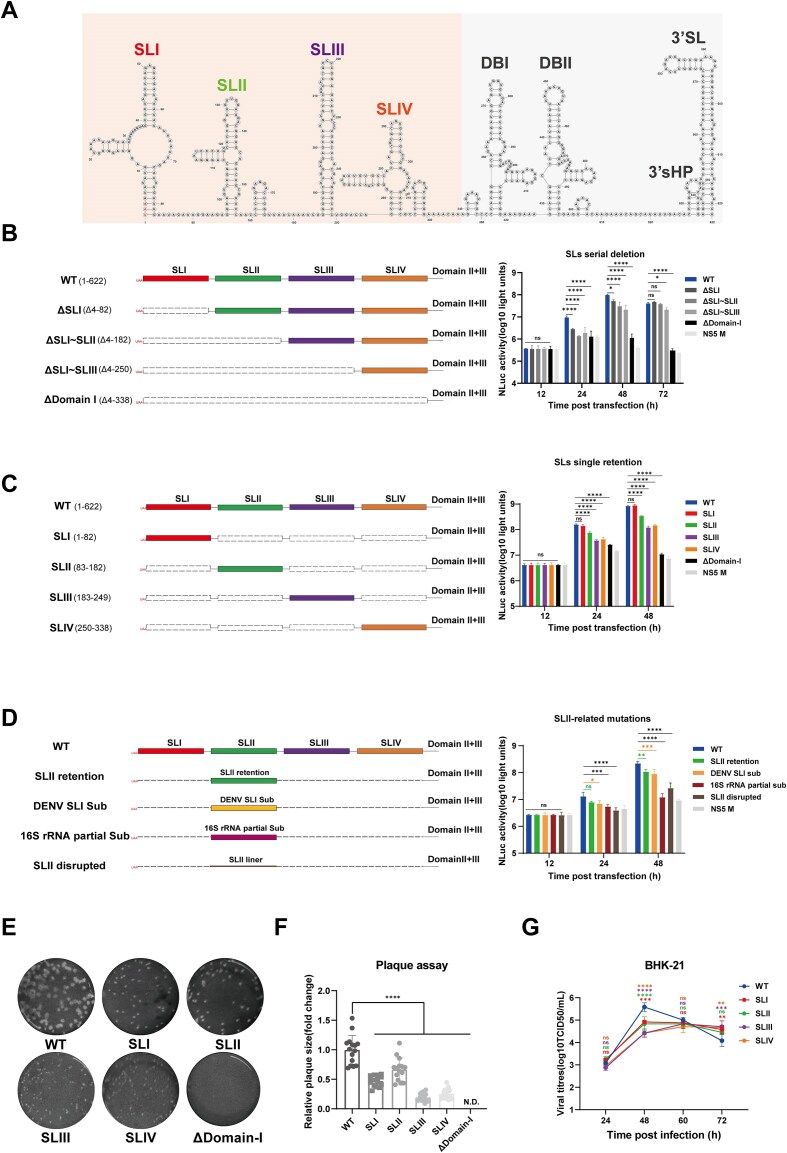
SL structures within 3′UTR Domain I are required for TMUV replication. (**A**) RNA secondary structure prediction of the TMUV CQW1 3′UTR. Domain I contains four SL structures (SLI to SLIV). (**B**) Schematic diagram of the SL deletion series (left panel), with the NS5 stop codon highlighted in red, and kinetics of NLuc activity produced by WT and mutant replicons in BHK-21 cells (right panel). A replication-defective replicon with an inactivated NS5 GDD motif (NS5 M) was used as a negative control. (**C**) Schematic diagram of single-SL-retention mutants (left panel) and kinetics of NLuc activity in BHK-21 cells (right panel). (**D**) Schematic diagram of SLII-retention mutants (left panel) and kinetics of NLuc activity in BHK-21 cells (right panel). Replicon plasmids were transfected into BHK-21 cells and, at the indicated time points, cells were harvested, lysed, and assayed for NLuc activity. (**E** and **F**) Plaque morphology of SL-retention viruses in BHK-21 cells at 6 dpi. Ten plaques per virus were randomly selected, and plaque sizes were measured using ImageJ software. (**G**) Growth kinetics of WT and SL-retention viruses in BHK-21 cells infected with 200 TCID_50_ of each virus. Supernatants were harvested at the indicated time points and titrated in BHK-21 cells. Data are presented as means ± SD. Statistical significance of plaque size differences was evaluated by one-way ANOVA, whereas two-way ANOVA was used for analysis of the replicon assay and growth curve data. *P < 0.05; **P < 0.01; ***P < 0.001; ****P < 0.0001; ns, not significant.

To investigate the role of SL structures in TMUV 3′UTR Domain I, we first predicted the RNA secondary structure of this region based on previous studies [2, 19, 28, 29] and illustrated it schematically (Fig. 1A). Because MBFVs exhibit divergent host adaptation strategies driven by structural variation in 3′UTR Domain I [12, 13, 15], we investigated the role of the four SL structures within this region of the TMUV genome. Replicon assays of mutants carrying serial deletions of these SLs showed a progressive decline in RNA replication, with complete removal of Domain I nearly abolishing replication in BHK-21 cells (Fig. 1B). To assess the contribution of individual SLs, we generated four single-SL-retention mutants. All of these mutants retained replication capacity to varying degrees, with the SLI-retention mutant displaying kinetics most similar to those of the WT in BHK-21 cells (Fig. 1C).

To determine whether viral replication depended on SL type or sequence length, we replaced SLII—the most prevalent structure among MBFVs—with the homologous DENV SLI, an exogenous SL derived from bacterial 16S rRNA, or a structure-disrupted SLII sequence (Supplementary Fig. S1A; Fig. 1D, left). Replicons containing DENV SLI showed replication comparable with that of the SLII-retention mutant, whereas those containing 16S rRNA or the disrupted SLII sequence exhibited only low-level replication in BHK-21 cells (Fig. 1D). These findings suggest that TMUV replication efficiency depends on the specific structural features of the retained SL element.

We then introduced the four single-SL-retention mutations and the ΔDomain I mutation into the TMUV icDNA clone to assess their effects on viral growth *in vitro*. Following transfection into BHK-21 cells, IFAs confirmed that all single-SL-retention viruses were viable. At 48, 72, and 96 hpt, the SLI- and SLII-retention mutants showed fluorescence signals comparable with those of the WT, whereas the SLIII-retention, SLIV-retention, and ΔDomain I mutants displayed markedly weaker fluorescence signals than the WT at all time points (Supplementary Fig. S2). Plaque assays in BHK-21 cells showed that all single-SL-retention mutants produced significantly smaller plaques than the WT, whereas the ΔDomain I mutant produced no detectable plaques (Fig. 1E, F). Analysis of growth kinetics in BHK-21 cells further demonstrated attenuated replication in all mutants. The SLI- and SLII-retention mutants reached peak titres at 48 hpi, similar to the WT, but their titres were 4.6- and 5.7-fold lower, respectively. By contrast, the SLIII- and SLIV-retention mutants exhibited delayed peak titres at 60 hpi and showed 5.7- and 7.5-fold reductions, respectively (Fig. 1G). Collectively, these findings indicate that although a single SL is sufficient to support TMUV RNA replication, efficient viral proliferation requires the coordinated presence of multiple SL structures within 3′UTR Domain I.

### SLII copy number modulates TMUV proliferation in diverse host cells

**Figure 2.**
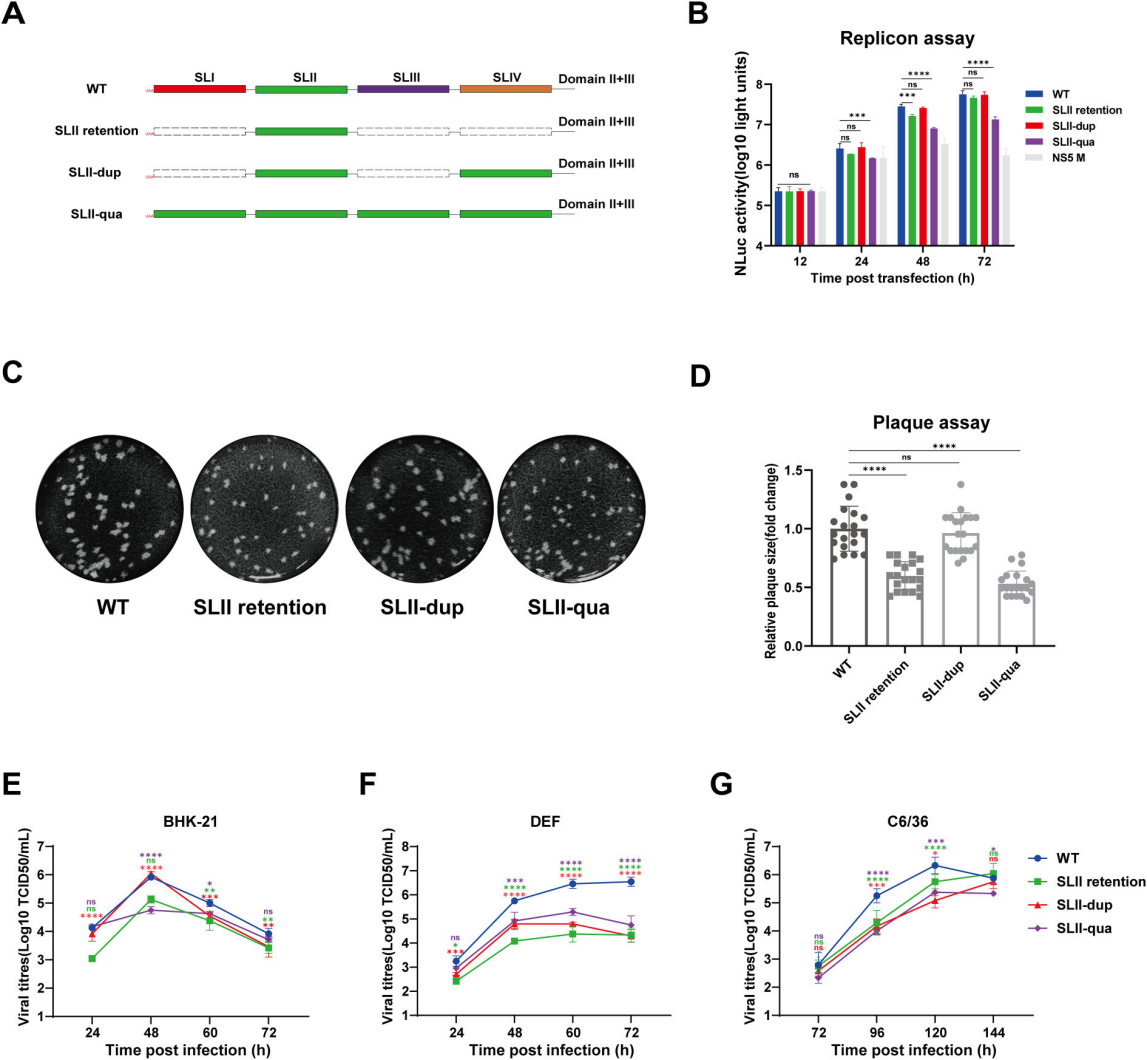
Specific duplication of SLII contributes to TMUV proliferation in avian cells. (**A**) Schematic diagram of mutants with SLII copy number variations. (**B**) NLuc expression kinetics of WT and mutant replicons in BHK-21 cells were assessed as described in the legend to Fig. 1B–D. (**C** and **D**) Plaque morphology of WT and mutant viruses in BHK-21 cells; 20 plaques per virus were randomly selected for plaque size measurement using ImageJ software. (**E**–**G**) Growth kinetics of WT and mutant viruses in BHK-21 (E), DEF (F), and C6/36 (G) cells infected with 200 TCID_50_ of the indicated viruses; supernatants were harvested at the indicated time points and titrated. Data represent the means ± SDs from three independent experiments. Statistical significance of plaque size differences was determined by one-way ANOVA, whereas significance of replicon assays and viral growth curves was determined by two-way ANOVA. *P < 0.05; **P < 0.01; ***P < 0.001; ****P < 0.0001; ns, not significant.

The 3′UTR Domain I of MBFVs contains variable numbers of SL structures; for example, DENV and ZIKV harbour two conserved SL elements, whereas WNV and TMUV contain additional related but non-identical SL copies. To assess whether the copy number of the conserved SLII element affects TMUV replication, we generated constructs in which Domain I contained one, two, or four copies of SLII, designated SLII-retention, SLII-dup, and SLII-qua, respectively (Fig. 2A). Replicon assays in BHK-21 cells showed that SLII-dup replicated with kinetics comparable with those of the WT, whereas SLII-qua was significantly attenuated (Fig. 2B). When these modifications were introduced into viral genomes, all mutant viruses were viable (Supplementary Fig. S3A). Plaque assays in BHK-21 cells further showed that SLII-dup produced plaques similar in size to those of the WT, whereas SLII-retention and SLII-qua generated significantly smaller plaques (Fig. 2C, D). Consistently, growth kinetics in BHK-21 cells demonstrated that SLII-dup reached WT-comparable titres at all time points, whereas SLII-retention and SLII-qua were significantly attenuated at 48 and 60 hpi (Fig. 2E). These results indicate that two copies of SLII are sufficient for efficient TMUV proliferation, whereas four copies suppress replication.

To determine whether variation in SLII copy number exerts host-specific effects, we evaluated viral replication in duck (DEF) and mosquito (C6/36) cells. In DEF cells, all mutants displayed impaired proliferation compared with the WT, with peak titres reduced by 158-fold (SLII-retention), 56-fold (SLII-dup), and 18-fold (SLII-qua) (Fig. 2F). In contrast, in C6/36 cells, SLII-retention and SLII-dup exhibited delayed proliferation but ultimately reached WT-level titres, whereas SLII-qua remained attenuated throughout infection (Fig. 2G).

Notably, the construct containing two SLII copies maintained replication in mosquito cells and restored replication to WT-like levels in mammalian cells, yet remained attenuated in avian cells. The construct containing four SLII copies showed partial recovery of replication in avian cells but remained attenuated in both mammalian and mosquito cells. Together, these findings suggest that structurally distinct SL configurations within TMUV Domain I may fine-tune viral replication across diverse host environments.

### Different SL structures in Domain I have contrasting functions in TMUV proliferation across diverse host cells

**Figure 3.**
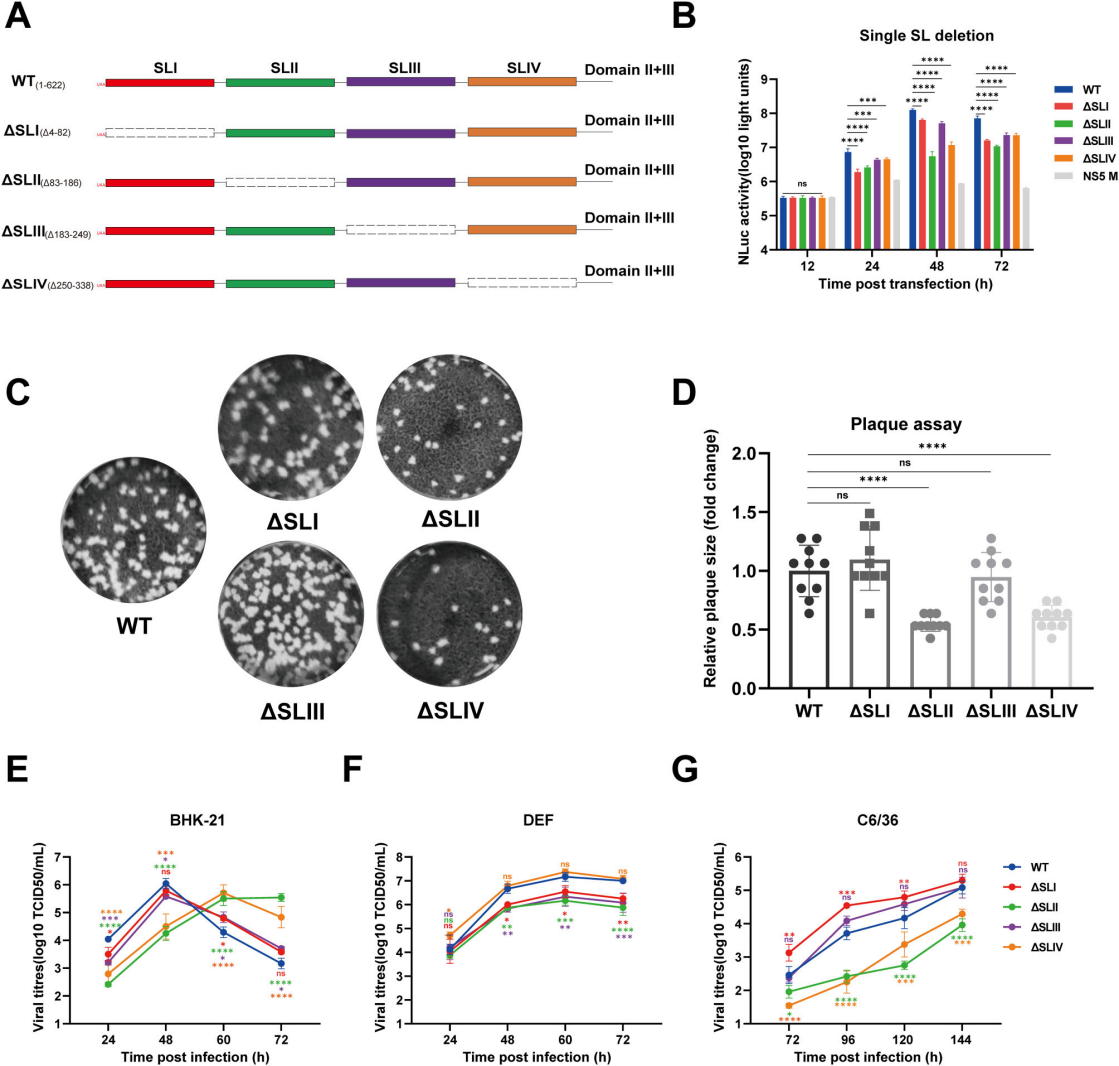
Different SL structures in the TMUV 3′UTR Domain I have cell type-specific functions. (**A**) Schematic representation of single-SL-deletion mutants. (**B**) NLuc expression kinetics of WT and mutant replicons in BHK-21 cells were assessed as described in the legend to Fig. 1B–D. (**C** and **D**) Plaque morphology of WT and mutant viruses in BHK-21 cells (C); 10 plaques per virus were randomly selected and measured using ImageJ software (D). (**E–G**) Growth kinetics of WT and mutant viruses in BHK-21 (E), DEF (F), and C6/36 (G) cells infected with 200 TCID_50_ of the indicated viruses. Data represent the means ± SDs of three independent experiments. A one-way ANOVA test was used to evaluate the statistical significance of plaque sizes, and a two-way ANOVA test was used to assess the significance of replicon assays and viral growth curves. *P < 0.05; **P < 0.01; ***P < 0.001; ****P < 0.0001; ns, not significant.

To test the hypothesis that TMUV may utilize distinct SL elements to support proliferation in different hosts, we constructed four single-SL deletion mutants (ΔSLI, ΔSLII, ΔSLIII, and ΔSLIV) (Fig. 3A) and evaluated their proliferation in diverse host cell types. Replicon assays in BHK-21 cells revealed reduced RNA replication in all deletion mutants; however, the extent of reduction differed markedly. At 48 hpt, ΔSLI and ΔSLIII showed only modest reductions in NLuc activity (∼2-fold relative to the WT), whereas ΔSLII and ΔSLIV exhibited much more pronounced decreases (∼23-and ∼12-fold, respectively) (Fig. 3B).

All corresponding viruses were successfully rescued (Supplementary Fig. S3). Consistent with the results of the replication assay, plaque assays in BHK-21 cells showed that ΔSLI and ΔSLIII formed plaques comparable in size with those of the WT, while ΔSLII and ΔSLIV produced significantly smaller plaques (Fig. 3C, D). Growth curve analysis further demonstrated that ΔSLI and ΔSLIII reached WT-like peak titres at 48 hpi, whereas ΔSLII and ΔSLIV exhibited delayed peaks at 60 hpi (Fig. 3E). In DEF cells, however, ΔSLIV replicated similarly to the WT, whereas all other mutants showed reduced growth (Fig. 3F). In C6/36 cells, ΔSLI replicated slightly faster than the WT, ΔSLIII showed WT-like replication, and ΔSLII and ΔSLIV were significantly attenuated (Fig. 3G).

Taken together, these results indicate that SLII facilitates viral proliferation in all tested cell types. By contrast, the other SL structures appear to have distinct cell-type-specific roles. SLI and SLIII were dispensable in BHK-21 and C6/36 cells but were important for viral proliferation in DEF cells. Conversely, SLIV was non-essential in DEF cells but contributed to replication in both BHK-21 and C6/36 cells.

To further assess whether SLI and SLIII remain stable during host-restricted replication, we serially passaged WT TMUV in BHK-21 cells for 10 passages and in C6/36 cells for five passages, followed by next-generation sequencing analysis of the viral 3′UTR (Supplementary Table S2). No major changes in the 3′UTR, such as deletion of SL structures, were observed under either passage condition. In C6/36 cells, no SNPs were detected within the SL regions. In BHK-21 cells, three SNPs were identified, including two in SLI and one in SLIII, but none was predicted to alter RNA secondary structure. These findings indicate that SLI and SLIII are not readily lost during short-term passage in either mammalian or mosquito cells.

### SLI and SLIII specifically promote TMUV proliferation in avian but not mammalian cells

**Figure 4.**
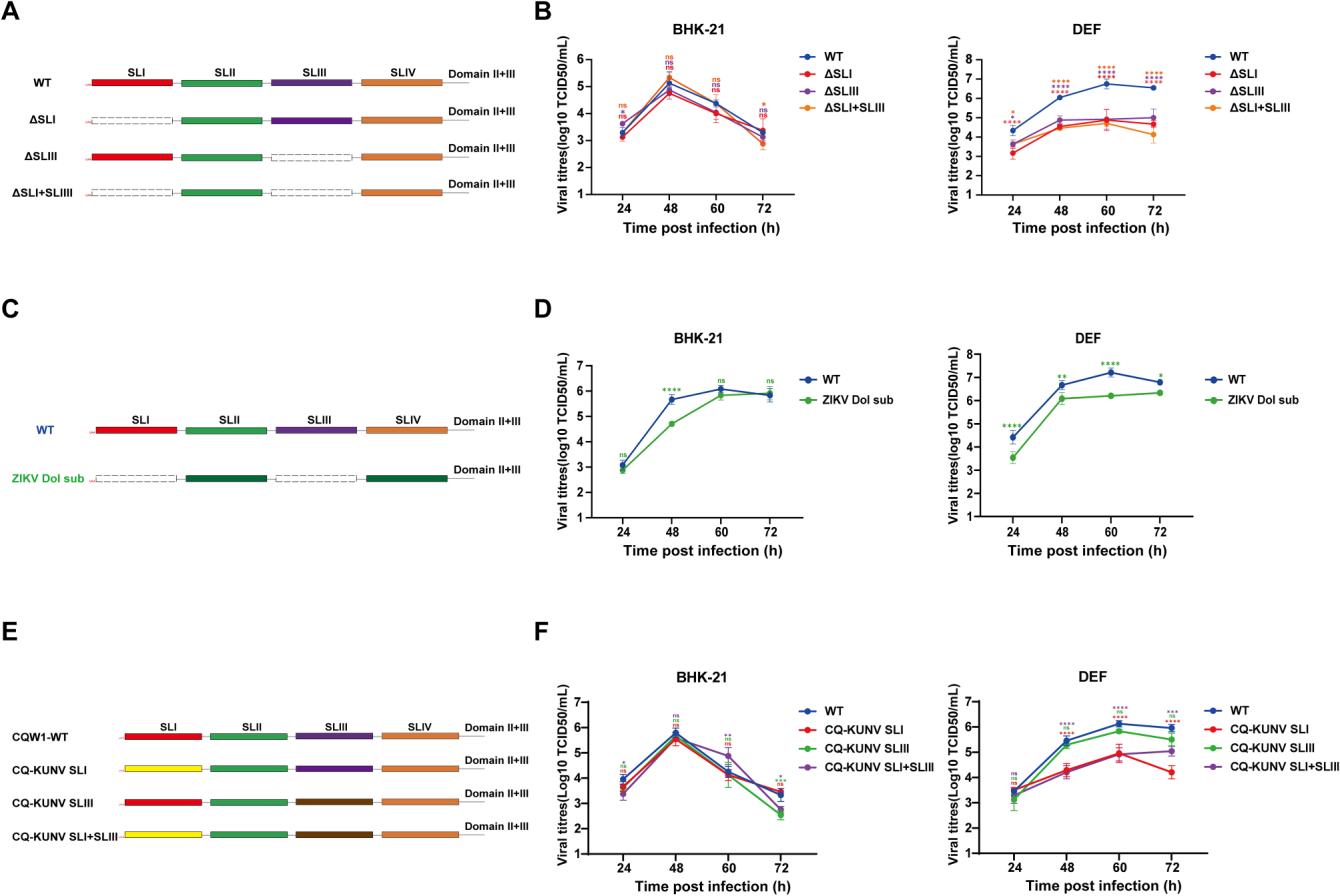
SLI and SLIII are essential for TMUV replication in avian cells. (**A**) Schematic representation of SLI and/or SLIII deletion mutants. (**B**) Growth kinetics of WT and mutant viruses in BHK-21 (left panel) and DEF (right panel) cells infected with 200 TCID_50_ of the indicated viruses. (**C**) Schematic representation of ZIKV Domain I substitutions. (**D**) Growth kinetics of WT TMUV and chimeric viruses in BHK-21 (left panel) and DEF (right panel) cells infected with 200 TCID_50_ of the indicated viruses. (**E**) Schematic representation of KUNV SLI and/or SLIII substitutions. (**F**) Growth kinetics of WT TMUV and chimeric viruses in BHK-21 (left panel) and DEF (right panel) cells infected with 200 TCID_50_ of the indicated viruses. Data represent the means ± SDs of three independent experiments. A two-way ANOVA test was used to assess the statistical significance of viral growth curves. *P < 0.05; **P < 0.01; ***P < 0.001; ****P < 0.0001; ns, not significant.

To further investigate the significance of SLI and SLIII for viral fitness in avian cells, we generated a ΔSLI + SLIII mutant of TMUV (Fig. 4A). In BHK-21 cells, the corresponding replicon showed only a modest (∼2-fold) reduction in NLuc activity at 48 hpt, and this difference was no longer detectable at 72 hpt (Supplementary Fig. S4A). The corresponding virus was successfully rescued (Supplementary Fig. S3A), and in BHK-21 cells, no difference in plaque size compared with the WT TMUV was observed (Supplementary Fig. S4B). Consistently, its growth kinetics in BHK-21 cells were also comparable with those of the WT; in contrast, replication of the ΔSLI + SLIII mutant in DEF cells was reduced at all time points (Fig. 4B). These results indicate that SLI and SLIII enhance TMUV proliferation in DEF cells but are non-essential in BHK-21 cells.

To validate this observation, we substituted TMUV Domain I with the homologous region from ZIKV, which naturally lacks SLI and SLIII structures (Fig. 4C). In BHK-21 cells, the chimeric replicon reached WT-like peak NLuc activity at 48 hpt (Supplementary Fig. S4C), and the rescued virus showed no difference in plaque size compared with the WT (Supplementary Fig. S4D). Growth kinetics revealed only slightly reduced replication in BHK-21 cells at 48 hpi, and titres became comparable with those of the WT at later time points (60–72 hpi). In DEF cells, however, the ZIKV Domain I chimera was significantly attenuated throughout infection (Fig. 4D).

Together, these results indicate that intact SLI and SLIII are required for efficient replication in DEF cells. Combined with the findings that the presence of four SLII copies only partially restored TMUV replication in DEF cells (Fig. 2F), this suggests that properly structured SLI and SLIII contribute to efficient viral proliferation in avian cells. To test this, we substituted TMUV SLI and/or SLIII with homologous structures from the KUNV FLSDX strain, generating CQ-KUNV SLI, CQ-KUNV SLIII, and CQ-KUNV SLI + SLIII (Fig. 4E). Sequence and RNA structure analyses showed that SLI differs substantially between TMUV and KUNV in both sequence and structure, whereas SLIII is more structurally conserved despite sequence divergence (Supplementary Fig. S5). All chimeric viruses were successfully rescued (Supplementary Fig. S3A), and in BHK-21 cells they produced plaques comparable with those of the WT (Supplementary Fig. S4E) as well as reaching WT TMUV-like peak titres at 48 hpi. In DEF cells, only CQ-KUNV SLIII showed WT TMUV-like replication, whereas CQ-KUNV SLI and CQ-KUNV SLI + SLIII were significantly attenuated (Fig. 4F). These findings support our conclusion that proper SLI and SLIII structures are important for TMUV replication in DEF cells, while having minimal impact in BHK-21 cells.

### Deletion of SLI and SLIII attenuates TMUV virulence

**Figure 5.**
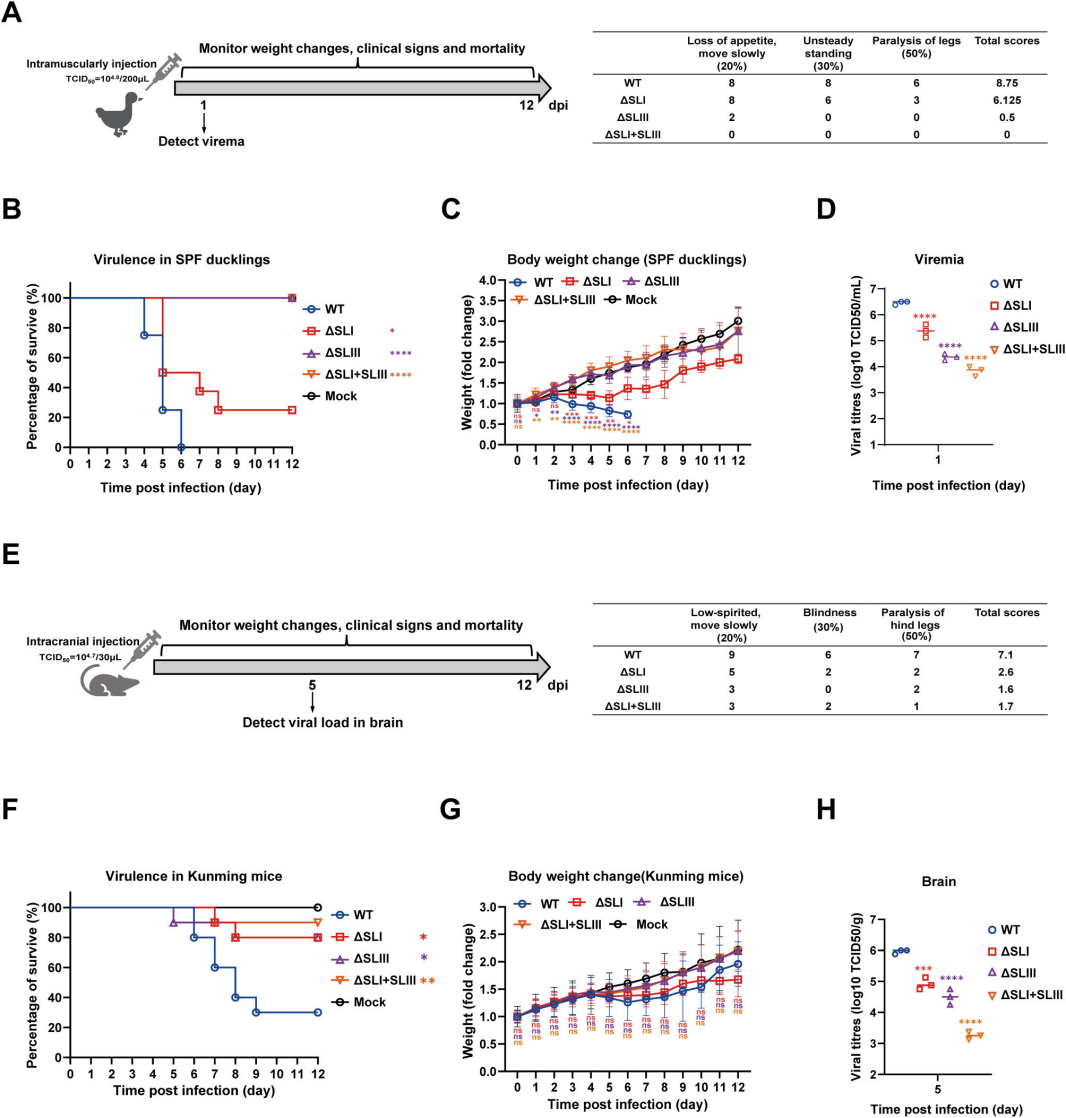
SLI and SLIII are important for TMUV virulence *in vivo*. (**A**) Experimental design (left panel) for SPF duckling experiments: 5-day-old SPF ducklings (*n* = 8 per group) were intramuscularly injected with WT, ΔSLI, ΔSLIII, or ΔSLI + SLIII TMUV at 10^4.8^ TCID_50_; ducklings in the mock group received DMEM. Weight changes, clinical signs, and mortality were monitored daily; clinical score points (right panel) were calculated as 20% × item 1 + 30% × item 2 + 50% × item 3. (**B**) Survival curves of infected ducklings. (**C**) Weight changes of infected ducklings from 0 to 12 dpi. (**D**) Viraemia levels of infected ducklings (*n* = 3 per group), with sera harvested at 1 dpi and titrated on BHK-21 cells. (**E**) Experimental design (left panel) for mouse experiments: 14-day-old Kunming mice (*n* = 10 per group) were intracranially injected with WT, ΔSLI, ΔSLIII, or ΔSLI + SLIII TMUV at 10^4.7^ TCID_50_; the mock group received DMEM. Weight changes, clinical signs, and mortality were monitored daily, and clinical score points (lower panel) were calculated as above. (**F**) Survival curves of infected mice. (**G**) Weight changes of infected mice from 0 to 12 dpi. (**H**) Viral loads in the brains of infected mice (*n* = 3 per group). Brains were harvested at 5 dpi, homogenized in PBS, and titrated on BHK-21 cells. Data represent the means ± SDs. Statistical significance of survival was analysed using the log-rank (Mantel–Cox) test; body weight changes were analysed using multiple *t*-tests with the Holm–Sidak method; viraemia levels were analysed using two-way ANOVA; and viral loads in mouse brains were analysed using one-way ANOVA. *P < 0.05; **P < 0.01; ***P < 0.001; ****P < 0.0001; ns, not significant.

To determine whether the host-specific effects observed in cell culture were also reflected *in vivo*, we evaluated the impact of SLI and SLIII structures on TMUV virulence. First, we used 5-day-old SPF ducklings, a highly susceptible model for TMUV pathogenesis (Fig. 5A). Clinical scores were lower in all mutant-infected groups than in the WT-infected group. However, clear differences were observed among the mutants: ΔSLI-infected ducklings developed typical clinical signs of TMUV infection, including unsteady posture and leg paralysis, whereas ΔSLIII- and ΔSLI + SLIII-infected animals showed only mild symptoms (Fig. 5A). Specifically, WT-infected ducklings began losing weight at 3 dpi and reached 100% mortality, and ΔSLI-infected animals showed weight loss from 4 dpi and 75% mortality. In contrast, ΔSLIII- and ΔSLI + SLIII-infected ducklings exhibited only slight weight loss and no mortality during the observation period (Fig. 5B, C). At 1 dpi, serum viral loads were 10^6.46^ and 10^5.38^ TCID_50_ ml^−1^ in WT- and ΔSLI-infected ducklings, respectively, whereas ΔSLIII- and ΔSLI + SLIII-infected groups showed significantly lower titres of 10^4.38^ and 10^3.83^ TCID_50_ ml^−1^, respectively (Fig. 5D).

Next, to determine whether attenuation was specific to avian hosts, we assessed the virulence of the mutant viruses in 14-day-old Kunming mice (Fig. 5E). No significant differences in body weight changes were observed across groups (Fig. 5G). However, 70% of WT-infected mice developed severe clinical signs by 5 dpi, including blindness and hind-limb paralysis. Similar symptoms were observed in 40, 30, and 30% of mice in the ΔSLI-, ΔSLIII-, and ΔSLI + SLIII-infected groups, respectively (Fig. 5E). Mortality mirrored these outcomes: 70% of WT-infected mice died compared with 20, 20, and 10% of ΔSLI-, ΔSLIII-, and ΔSLI + SLIII-infected mice, respectively (Fig. 5F). Consistently, viral titres in the brain at 5 dpi were significantly higher in WT TMUV-infected animals (10^5.96^ TCID_50_ g^−1^) than in mutant-infected animals (ΔSLI: 10^4.92^ TCID_50_ g^−1^, ΔSLIII: 10^4.67^ TCID_50_ g^−1^, ΔSLI + SLIII: 10^3.25^ TCID_50_ g^−1^) (Fig. 5H).

Together, these findings demonstrate that deletion of SLI and SLIII attenuates TMUV virulence *in vivo*. The contribution of each element, however, differed between host models: in ducklings, attenuation was driven predominantly by loss of SLIII, with only a modest additional effect when both elements were deleted, whereas in mice the effects of SLI and SLIII deletion were less clearly differentiated. Overall, attenuation of pathogenicity was more pronounced in ducklings than in mice.

### JEV uses structural strategies similar to those of TMUV for proliferation to avian hosts

JEV is an MBFV primarily transmitted by *Culex* mosquitoes and, like TMUV, uses birds as reservoir or amplification hosts. In 1954, a mosquito-derived genotype III (GIII) JEV isolate, designated SA14, was isolated from *Culex pipiens* in Xi’an, China [30]. More recently, a duck-derived genotype I (GI) JEV isolate, duck/2022-SD-1 (SD-1), was isolated from Shandong Province, China [9, 31].

**Figure 6.**
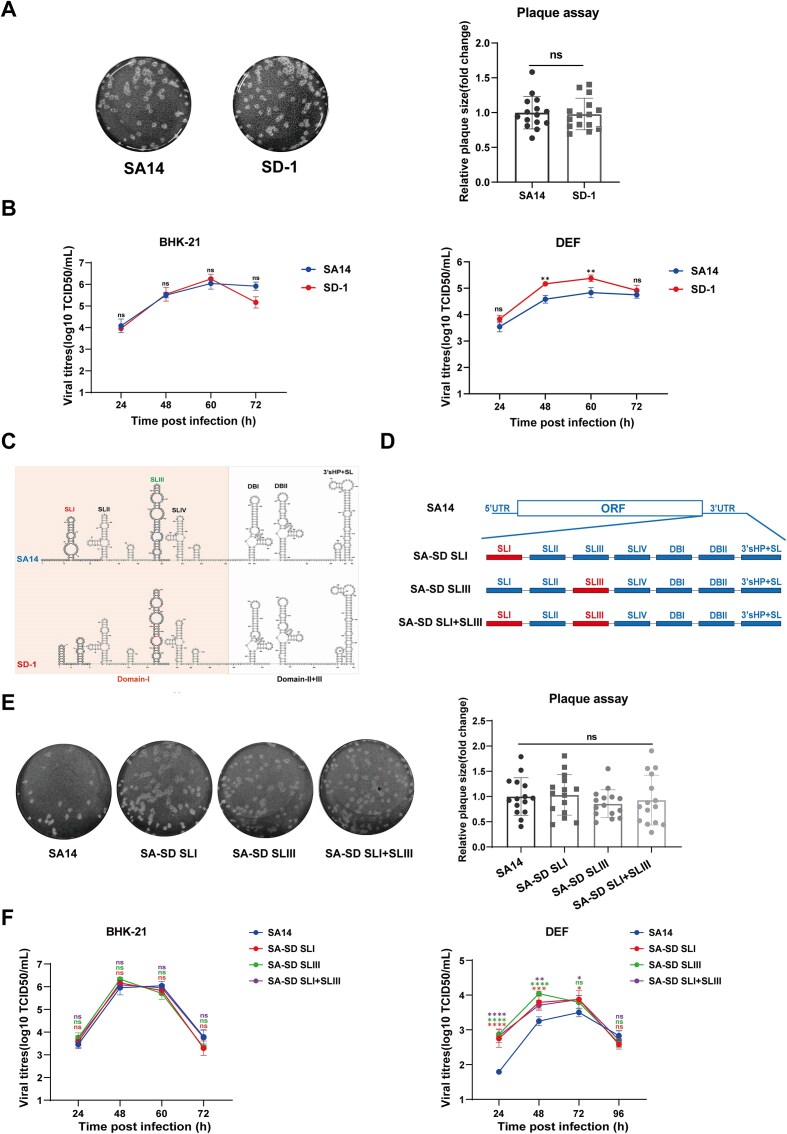
SLI and SLIII of a duck-derived JEV strain enhance proliferation of mosquito-derived JEV in DEF cells. (**A**) Plaque morphology of JEV SA14 and SD-1 isolates in BHK-21 cells, with 15 plaques per virus randomly selected and measured using ImageJ software; statistical significance was evaluated by Student’s *t*-test. (**B**) Growth kinetics of SA14 and SD-1 in BHK-21 (left panel) and DEF (right panel) cells infected with 200 TCID_50_ of virus. Data represent the means ± SDs of three independent experiments. Statistical significance was assessed by two-way ANOVA. (**C**) RNA secondary structure comparison of the 3′UTRs of SA14 (top) and SD-1 (bottom). SLI and SLIII structures are highlighted, and nucleotide differences in SLIII are shown in blue (SA14) and red (SD-1). (**D**) Schematic diagram of chimeric viruses generated using SA14 as the backbone. (**E**) Plaque morphology of SA14, SA-SD SLI, SA-SD SLIII, and SA-SD SLI + SLIII viruses in BHK-21 cells, with 15 plaques per virus randomly selected and measured using ImageJ software; statistical significance was assessed by one-way ANOVA. (**F**) Growth kinetics of SA14, SA-SD SLI, SA-SD SLIII, and SA-SD SLI + SLIII viruses in BHK-21 (left panel) and DEF (right panel) cells infected with 200 TCID_50_ of virus; statistical significance was evaluated by two-way ANOVA. Data represent the means ± SDs of three independent experiments. *P < 0.05; **P < 0.01; ***P < 0.001; ****P < 0.0001; ns, not significant.

Comparative analysis showed that SD-1 produced plaques similar in size to those of SA14 and displayed comparable replication kinetics in BHK-21 cells (Fig. 6A), whereas in DEF cells it exhibited enhanced replication (Fig. 6B). To determine whether the 3′UTR of SD-1 contributes to avian adaptation of JEV, we constructed a chimeric virus (SA-SD 3′UTR) by replacing the SA14 3′UTR with that of SD-1 (Supplementary Fig. S6A). The chimeric virus was successfully rescued (Supplementary Fig. S3B) and, in BHK-21 cells, showed plaque morphology and replication kinetics comparable with those of SA14 (Supplementary Fig. S6B–D). In DEF cells, however, it exhibited significantly higher titres than SA14 (Supplementary Fig. S6E). These results indicated that the 3′UTR of SD-1 facilitates JEV proliferation in avian cells.

To further identify whether SLI and SLIII are responsible for the observed phenotypic difference, we compared the RNA secondary structures of SLI and SLIII in the SA14 and SD-1 isolates. We found that SLI showed poor secondary structure conservation, whereas the SLIII structures of the two strains were similar and their sequences differed only by six point-mutations (Fig. 6C). The sequence differences between the SLIII structures of the mosquito- and duck-derived JEV genotypes preserve the predicted secondary structure. This compensatory variation provides important evidence supporting the functional relevance of this RNA element and suggests that structural conservation is maintained despite sequence divergence. Guided by these data, we constructed and rescued three chimeric viruses: SA-SD SLI, SA-SD SLIII, and SA-SD SLI + SLIII (Fig. 6D; Supplementary Fig. S3B), and evaluated their replication. In BHK-21 cells, all chimeras formed plaques similar to those of WT SA14 and showed growth kinetics comparable with those of WT SA14 (Fig. 6E, F). In DEF cells, however, all three chimeras exhibited enhanced proliferation compared with WT SA14 (Fig. 6F). Together with the TMUV data (Fig. 4), these findings indicate that specialized SLI and SLIII configurations contribute to proliferation of both TMUV and JEV in avian hosts.

### Deletion of SLI and SLIII attenuates TMUV replication independently of viral genome translation, packaging, or altered interferon-β induction

**Figure 7.**
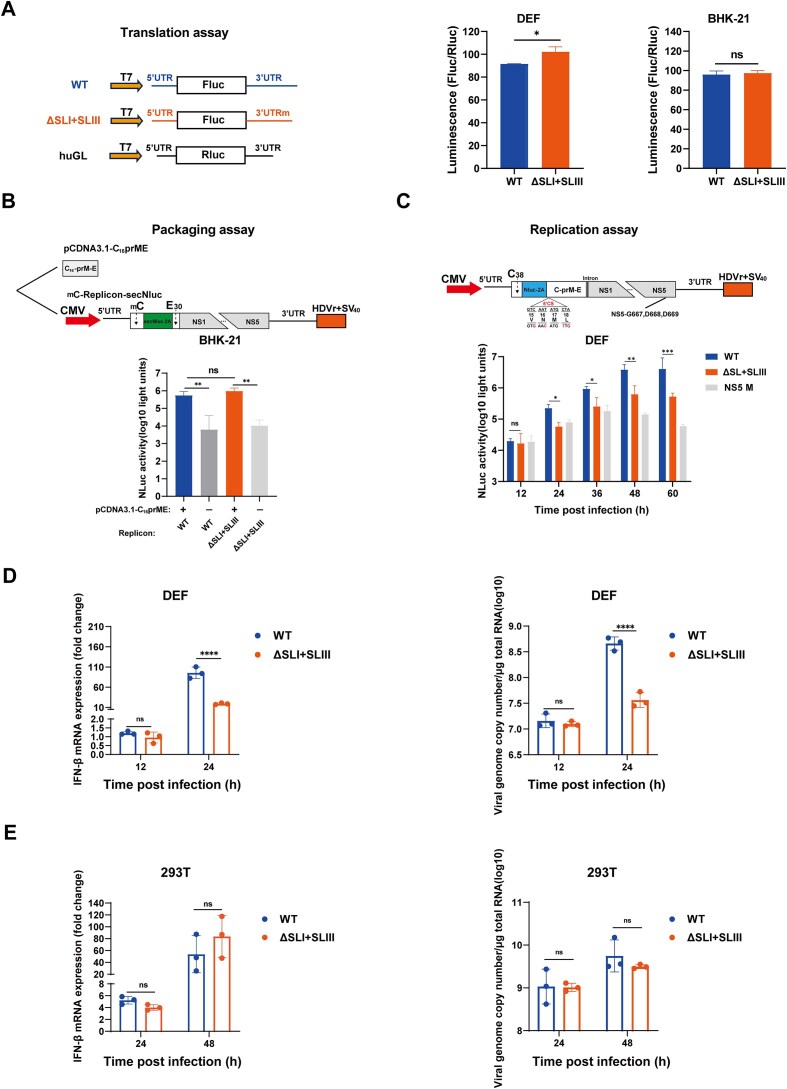
SLI and SLIII regulate TMUV genome replication. (**A**) Schematic diagram of the constructs used for *in vitro* transcription (left panel), and translation efficiencies of mRNAs harbouring the WT or ΔSLI + SLIII 3′ UTR in DEF (middle panel) and BHK-21 cells (right panel). Transfected cells were lysed at 6 hpt, reporter activities were measured, and FLuc activity was normalized to RLuc activity. (**B**) Schematic diagram of the packaging system (upper panel), and the results of the packaging assay (lower panel). BHK-21 cells were co-transfected with WT or ΔSLI + SLIII replicon plasmids together with the C_16_prME packaging plasmid or without it (negative control). The supernatants containing single-round infectious particles (SRIPs) were used to infect fresh BHK-21 cells. At 24 hpi, the cells were lysed and NLuc activity was measured. (**C**) Schematic diagram of the TMUV reporter virus production construct (upper panel), and the kinetics of NLuc activity produced by WT and mutant reporter viruses to assess their genome replication in DEF cells. NLuc expression was assessed as described in the legend to Fig. 1B–D. (**D**) DEF cells were infected with 10^4^ TCID_50_ per well. At the indicated time points, the copy number of intracellular viral genomic RNA (left panel) and IFN-β mRNA (right panel) was determined by RT–qPCR. IFN-β mRNA levels in infected cells were normalized to those in mock-infected control cells using β-actin mRNA as a reference. (**E**) HEK-293T cells were infected with 10^4^ TCID_50_ per well. At the indicated time points, the copy number of intracellular viral genomic RNA (left panel) and IFN-β mRNA (right panel) was determined by RT–qPCR. IFN-β mRNA levels in infected cells were normalized to those in mock-infected control cells using GAPDH mRNA as reference. Data represent the means ± SDs of three independent experiments. The statistical significance of viral titres, viral genome copy numbers, and IFN-β mRNA expression in DEF cells was analysed using one-way ANOVA; the translation efficiency of *in vitro* transcribed RNAs was assessed using Student’s *t*-test. *P < 0.05; **P < 0.01; ***P < 0.001; ****P < 0.0001; ns, not significant.

The orthoflavivirus 3′UTR has been implicated in translation, genome replication, and virion assembly [19, 32, 33]. To elucidate the mechanism by which SLI and SLIII affect host-specific TMUV proliferation, we examined the effects of their deletion on these stages of the viral life cycle. Deletion of SLI and SLIII had only a slight impact on translation (Fig. 7A) or packaging (Fig. 7B). Because the TMUV replicon system is non-functional in DEF cells [27], we adopted a strategy similar to that used for ZIKV [13] and constructed a TMUV reporter virus that allowed assessment of genome replication in a replicon-like manner (Fig. 7C). Using this system, we found that simultaneous deletion of SLI and SLIII markedly impaired TMUV genome replication in DEF cells, as indicated by an ~7-fold reduction in NLuc activity at 48 hpt and a 10-fold reduction at 60 hpt (Fig. 7C). In contrast, the ΔSLI + SLIII mutant replicon exhibited only a mild (∼2-fold) decrease in NLuc activity in BHK-21 cells at 48 hpt, and this difference was no longer apparent by 72 hpt (Supplementary Fig. S4A). These results indicate that deletion of SLI and SLIII selectively attenuates viral genomic RNA replication in avian cells, with only limited and transient effects in BHK-21 cells.

Highly structured SL elements in the 3′UTR of flaviviruses are known to contribute to sfRNA production, which can antagonize IFN responses and thereby affect viral proliferation [15, 34, 35]. Because BHK-21 cells are deficient in type I interferon (IFN-I) production and signalling, whereas DEF cells retain IFN responsiveness, we considered the possibility that differential IFN-I induction might contribute to the distinct replication phenotypes of WT and ΔSLI + SLIII viruses. To address this, we measured IFN-β mRNA levels in DEF and HEK-293T cells following infection. In DEF cells, both viruses induced IFN-β, but WT infection yielded ~6-fold higher levels than the mutant at 24 hpi, probably reflecting more efficient genome replication (Fig. 7D). In HEK-293T cells, WT and mutant viruses replicated similarly and induced comparable levels of IFN-β mRNA (Fig. 7E). Together, these findings suggest that the host-specific effect of SLI and SLIII on TMUV proliferation is consistent with the effect of SLI/SLIII on proliferation being independent of altered IFN-β induction.

### CELF2 is an SLI/SLIII-associated host factor that promotes TMUV proliferation in a host-specific manner

**Figure 8.**
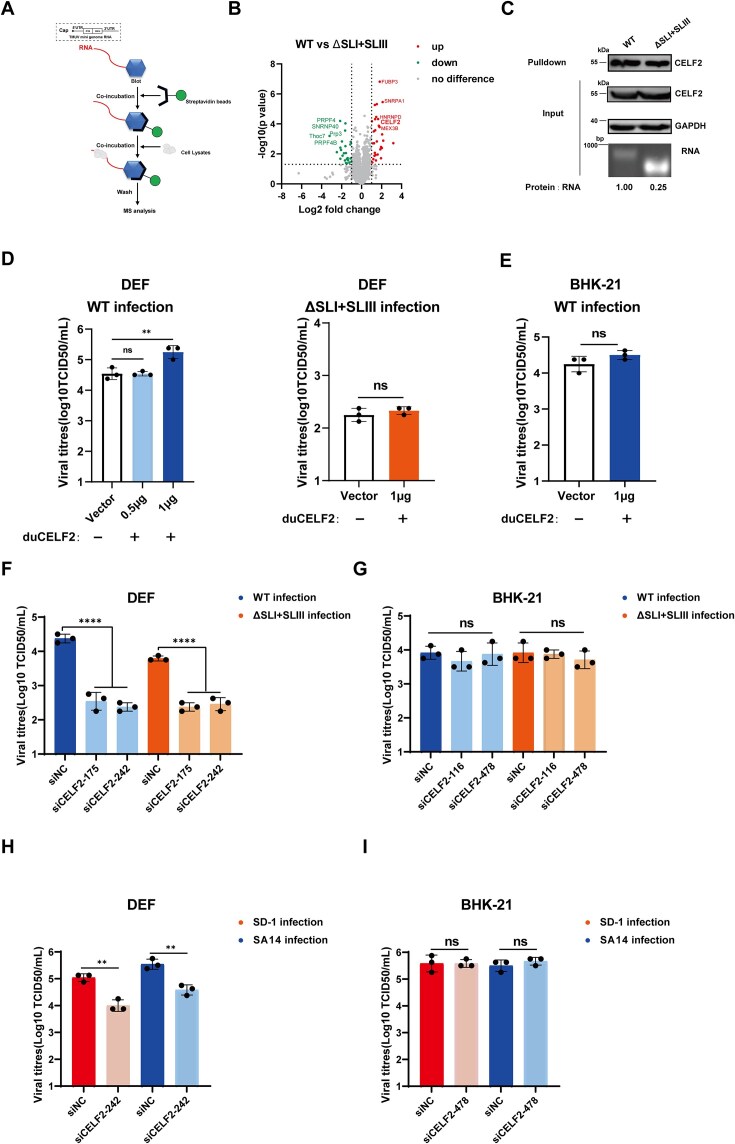
CELF2 promotes TMUV proliferation in DEF cells. (**A**) Schematic diagram of the RNA–protein complex pulldown assay. (**B**) Blot diagram of proteins differentially associated with WT and ΔSLI + SLIII mutant RNAs. Proteins significantly decreased relative to the WT are highlighted in green, whereas those significantly increased are highlighted in red. Values represent the means of three independent experiments. (**C**) *In vitro* transcribed, biotinylated WT or ΔSLI + SLIII mutant RNAs were incubated with extracts from DEF cells transfected with a plasmid expressing duCELF2-Flag, and RNA–protein complexes were captured on streptavidin beads. RNA input was assessed by electrophoresis on 1% formaldehyde-denatured agarose gels. Proteins were detected using antibodies against Flag and GAPDH, the latter serving as a loading control. The ratio of pulled-down duCELF2 to input biotinylated RNA was quantified using ImageJ software. (**D**) DEF cells were transfected with the indicated amounts of duCELF2-Flag expression plasmid and infected at 36 hpt with TMUV WT or ΔSLI + SLIII (10^4^ TCID_50_) for 36 h, and viral titres in the supernatants were determined. (**E**) At 36 hpt, BHK-21 cells transfected with 1 μg of duCELF2-Flag expression plasmid or empty vector were infected with TMUV WT (10^4^ TCID_50_). Viral titres in the supernatants were determined at 36 hpi. DEF cells (**F**) or BHK-21 cells (**G**) were transfected with 5 pmol of CELF2-specific siRNAs or non-specific control RNA (siNC) for 36 h and subsequently infected with TMUV WT or ΔSLI + SLIII (10^4^ TCID_50_) for 36 h; viral titres in the supernatants were determined. DEF cells (**H**) or BHK-21 cells (**I**) were transfected with 5 pmol of CELF2-specific siRNA or siNC for 36 h and subsequently infected with JEV SD-1 or SA14 (10^4^ TCID_50_) for 36 h. Viral titres in the supernatants were determined. Data represent the means ± SDs of three independent experiments. The effect of CELF2 overexpression on TMUV WT proliferation in DEF cells and the effect of CELF2 knockdown on TMUV proliferation in BHK-21 and DEF cells were analysed using one-way ANOVA; all other data were analysed by Student’s *t*-test. *P < 0.05; **P < 0.01; ***P < 0.001; ****P < 0.0001; ns, not significant.

The role of SLI/SLIII in host-specific TMUV genome replication implies the existence of host factors interacting with these elements, and that these factors or their impact differ between avian and mammalian cells. To identify such host factors involved in TMUV replication in DEF cells, we performed an RNA pulldown assay using biotinylated RNAs (Fig. 8A). LC-MS/MS analysis identified 134 duck proteins with differential binding to WT and ΔSLI + SLIII mutant RNAs, including 76 enriched and 58 reduced proteins (Fig. 8B; Supplementary Table S3). Among the enriched candidates, FUSE-binding protein 3 (FUBP3)—previously reported to promote JEV replication [36]—showed the greatest increase (fold change = 3.5, *P* = 1.53 × 10^−7^), followed by CUGBP Elav-like family member 2 (CELF2; fold change = 3.2, *P* = 4.78 × 10^−5^). Because CELF2 has not been implicated in orthoflavivirus infection, it was selected for further analysis.

RNA pulldown assays confirmed that WT RNA exhibited stronger binding to CELF2 than the ΔSLI + SLIII mutant RNA (Fig. 8C). To directly assess its functional role, we performed CELF2 overexpression and siRNA-mediated knockdown experiments and analysed their impact on TMUV replication; validation of overexpression and knockdown efficiency is shown in Supplementary Fig. S7. Overexpression of duck CELF2 (duCELF2) markedly enhanced TMUV-WT proliferation in DEF cells but had no detectable effect on the ΔSLI + SLIII mutant (Fig. 8D). In BHK-21 cells, duCELF2 overexpression resulted in only a modest increase in TMUV-WT infection (Fig. 8E), indicating a host-specific effect (Fig. 8E). Furthermore, in DEF cells, duCELF2 silencing reduced WT TMUV titres by ∼60-fold and ΔSLI + SLIII mutant titres by ∼25-fold (Fig. 8F). In contrast, hamster CELF2 (hamCELF2) silencing in BHK-21 cells had no significant effect on either virus (Fig. 8G). Consistently, CELF2 knockdown also significantly impaired replication of both SA14 and SD-1 JEV isolates in DEF cells (Fig. 8H) but not in BHK-21 cells (Fig. 8I). Collectively, these results identify CELF2 as an SLI/SLIII-associated proviral factor that promotes TMUV and JEV replication in a host-specific manner.

### SLI and SLIII elements are present in the 3′UTRs of avian-associated MBFVs

**Figure 9.**
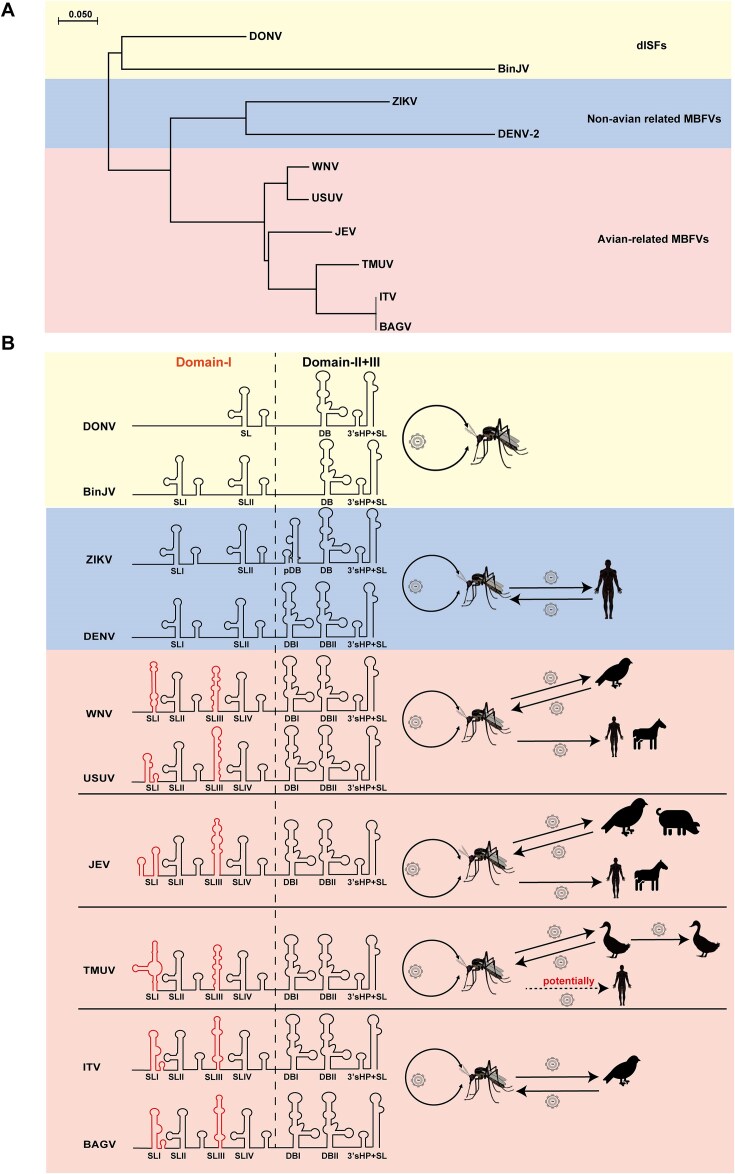
RNA secondary structure models within 3′UTR Domain I of mosquito-infecting flaviviruses. (**A**) Phylogenetic analysis of 3′UTR Domain I sequences. The phylogenetic tree was constructed using MEGA7 software with the Neighbor–Joining (NJ) method. dISFVs (DONV and BinJV) are highlighted in yellow, non-avian-related MBFVs (DENV and ZIKV) in blue, and avian-related MBFVs (WNV, USUV, JEV, TMUV, BAGV, and ITV) in pink. (**B**) RNA secondary structure predictions of 3′UTR Domain I correlate with viral transmission cycles. The avian-related MBFV-specific SLI and SLIII structures in Domain I are highlighted in red.

To place the findings that SLI and SLIII contribute to TMUV and JEV replication in avian hosts in a broader evolutionary context, we performed phylogenetic analysis of 3′UTR Domain I sequences from representative orthoflaviviruses, including dISFVs (DONV and BinjV), MBFVs that cycle between mosquitoes and mammals (DENV and ZIKV), and avian-associated MBFVs (WNV, USUV, TMUV, BAGV, ITV, and JEV). This analysis identified three distinct evolutionary clades (Fig. 9A).

RNA secondary structure prediction of Domain I in avian-associated MBFVs revealed conserved SL elements across this group. Compared with non-avian flaviviruses, avian-associated MBFVs possess two additional SL structures (SLI and SLIII) within Domain I. However, the sequences and folding patterns of these elements are not conserved among different avian-associated MBFVs (Fig. 9B).

Together, these findings suggest that SLI and SLIII act as common *cis-*acting elements that facilitate infection across diverse avian hosts despite structural variability.

## Discussion

A key feature of orthoflavivirus genomes is the presence of highly structured RNA duplications within 3′UTR Domain I, referred to as SL structures, which serve as a common mechanism for host-specific adaptation [28]. Evidence from multiple viruses supports this model: DONV, with a single SL structure, cannot replicate in mammalian cells, but acquires this ability when ZIKV-derived modules are introduced [1]. DENV and ZIKV contain two functionally relevant SL structures that regulate host-specific replication in mosquito cells [12, 13]. This phenomenon is not unique to mosquito-borne flaviviruses: a parallel case is also observed in chikungunya virus (CHIKV, an alphavirus), in which duplicated Y-shaped RNA structures promote replication in mosquito cells but reduce fitness in mammalian cells [37].

In this study, we demonstrate that TMUV harbours four distinct functional SL structures (SLI to SLIV) in 3′UTR Domain I that play divergent, host-specific roles in viral RNA replication. We show that both SL copy number and functional specialization contribute to TMUV proliferation in diverse host cells. In mosquito cells, retention of SLII alone is sufficient to support replication (Fig. 2G), consistent with previous findings for DENV and ZIKV. In contrast, efficient replication in mammalian cells requires either two SLII structures, the SLII and SLIV combination, or the presence of ZIKV Domain I (Figs 2E and 4C, D). However, increasing SL copy number without diversification—such as combining four identical SLII elements—reduces viral fitness (Fig. 2E), suggesting that both copy number and specialization contribute to viral host-specific proliferation. This principle is even more evident in avian cells: avian-associated MBFVs typically possess more complex and specific SL duplications; accordingly, we found that substitution of TMUV SLI and SLIII with homologous elements from KUNV significantly impaired replication in DEF cells (Fig. 4E, F). Importantly, analysis performed using JEV further supports this model. Genotype I strains, which have largely displaced genotype III in Asia and replicate more efficiently in birds [31, 38], carry SLI and SLIII elements that enhance replication when introduced into a GIII background in avian cells. Notably, although SLI and SLIII are common features of avian MBFVs, their sequences and structures are not conserved, suggesting independent evolutionary origins shaped by host-specific pressures.

The underlying mechanisms by which duplicated RNA structures contribute to viral host-specific adaptation have been partially elucidated, with sfRNA identified as one of the key influencing factors. In ZIKV, sfRNA-deficient mutants replicate efficiently in mosquito C6/36 cells but fail to establish sustained infection in mammalian A549 cells due to immune-mediated clearance [13, 34]. In DENV, *in vitro* host adaptation drives the accumulation of distinct sfRNA species in both vertebrate and invertebrate cells. Notably, point mutations selected in mosquito cells are sufficient to reshape sfRNA profiles, enhance IFN-I responses, and reduce viral fitness in human cells [39]. Collectively, these findings suggest that sfRNA plays a key role in host-specific adaptation by modulating host immune responses. Interestingly, our results showed that the WT and ΔSLI + SLIII mutant viruses exhibited similar replication capacities and induced comparable IFN-β production in mammalian cells (Fig. 7E), implying that the differences in their proliferation are largely independent of IFN induction. It has been shown that nucleotides upstream of SLs base-pair with nucleotides at the three-way junction to form an internal pseudoknot, which constitutes the structural basis of sfRNA formation [21, 40]. However, the SLI and SLIII structures in TMUV and JEV do not conform to these structural requirements for sfRNA formation. In contrast, SLII may be responsible for TMUV and JEV sfRNA production [41]. These observations suggest that the effects of SLI and SLIII on avian host adaptation in TMUV and JEV are unlikely to be explained primarily by sfRNA-related mechanisms and are therefore consistent with the existence of an additional mechanism.

RNA-binding proteins are also known to act as key determinants of host-specific adaptation in orthoflaviviruses [1, 42]. Several host proteins, including G3BP1 and SYNCRIP, have been shown to facilitate viral replication in mammalian hosts through interactions with the viral 3′UTR [1, 43]. In this study, specialized SLI and SLIII structures were found to enhance TMUV and JEV proliferation in avian cells (Figs 4 and 6). Using an RNA pulldown assay coupled with MS, we identified CELF2 as a previously unrecognized host factor associated with SLI/SLIII and demonstrated that duCELF2 promotes TMUV proliferation in DEF cells but not in BHK-21 cells (Fig. 8D–G). Notably, CELF2 also facilitates JEV proliferation in a host-specific manner (Fig. 8H, I), suggesting a broader role as a host factor enhancing orthoflavivirus RNA replication in avian cells. CELF2 is a multifunctional RNA-binding protein involved in multiple layers of gene regulation, including transcription, mRNA splicing, and mRNA stabilization [44]. Despite these established functions, its role in viral replication remains unexplored, and the molecular basis of its host-specific proviral activity therefore warrants further investigation.

Virulence of MBFVs is influenced by multiple determinants across the viral genome, including RNA secondary structures within the 3′UTR, which play important roles in pathogenesis [45]. Mutations, deletions, or substitutions of SL structures often attenuate virulence [46, 47, 48]. In TMUV, virulence determinants have been mainly attributed to the C protein [24, 49, 50], the E protein [51, 52, 53], and NS1 glycosylation [54], whereas the contribution of the 3′UTR has remained poorly understood. In this study, we identified SLI and SLIII as important determinants of TMUV pathogenicity in both SPF ducklings and suckling mice (Fig. 5). The severe attenuation observed upon their deletion highlights these structures as potential targets for vaccine development.

Collectively, our findings support a model in which, during the early stages of orthoflavivirus evolution, duplicated RNA elements may occasionally arise, potentially through RNA recombination [55]. These repeated elements may facilitate cross-species transmission and host-range expansion. Upon infection of a new host, selective pressures drive the functional specialization of duplicated elements to promote efficient replication and transmission, sometimes at the cost of reduced fitness in the original host. Therefore, this study provides new insights into the genetic determinants underlying virulence and avian host adaptation in MBFVs and offers a framework for rational vaccine design and strategies to limit viral spread.

## Supplementary Material

gkag791_Supplemental_File

## Data Availability

The raw data supporting the conclusions of this article will be made available by the authors, without undue reservation.
