# Supplementary material for: Duplication of specialized cis-acting elements SLI and SLIII in the viral RNA 3′UTR enables mosquito-borne orthoflavivirus adaptation to avian hosts
Source: Nucleic Acids Res. 2026 Aug 11;54(15):gkag791. doi: 10.1093/nar/gkag791 (PMC13458391; doi:10.1093/nar/gkag791)
Supplement: Supplementary file 1 [file gkag791_supplemental_file.pdf]

Supplementary Material

Duplication of specialized cis-acting elements SLI and SLIII in the viral RNA 3' UTR enables mosquito-borne flavivirus adaptation to avian hosts

Li Mao [1,†] , Yu He[1,2,3,4,5,†], Tao Wang[1,2,3,5,†], Zhen Wu[1,2,3,4,5], Andres Merits[6], Mingshu Wang [1,2,3,5] Renyong Jia[1,2,3,5], Dekang Zhu[1,2,3,5], Mafeng Liu[1,2,3,5], Xinxin Zhao[1,2,3,5], Qiao Yang[1,2,3,5], Ying Wu[1,2,3,5], Shaqiu Zhang[1,2,3,5], Juan Huang[1,2,3,5] , Xumin Ou[1,2,3,4,5], Di Sun[1,2,3,5], Bin Tian[1,2,3,4], Anchun Cheng [1,2,3,5,7,*] and Shun Chen [1,2,3,4,5,*]

1 Institute of Veterinary Medicine and Immunology, Sichuan Agricultural University, Chengdu, Sichuan 611130, China;

2 Research Center of Avian Disease, College of Veterinary Medicine, Sichuan Agricultural University, Chengdu, Sichuan 611130, China;

3 Agricultural Animal Diseases and Veterinary Public Health Key Laboratory of Sichuan Province, Sichuan Agricultural University, Chengdu, Sichuan 611130, China

4 Key Laboratory of Agricultural Bioinformatics, Ministry of Education, Sichuan Agricultural University, Chengdu, Sichuan 611130, China

5 Engineering Research Center of Southwest Animal Disease Prevention and Control Technology for Ministry of Education, Sichuan Agricultural University, Chengdu, Sichuan 611130, China

6 Institute of Bioenineering, University of Tartu, Tartu, Estonia

7 Institute of Veterinary Immunology and Green Drugs, Veterinary Department in College of Animal Science, State Key Laboratory of Green Pesticide, Guizhou University, Guiyang 550025, China

† Theses authors contributed equally to this article

* Correspondence: chenganchun@vip.163.com (A.C.); shunchen@sicau.edu.cn (S.C.); Tel.: +86-028-86291776

Contents:

Figure S1. RNA secondary structure prediction of elements used in recombinant TMUV Domain I.

Figure S2. Immunofluorescence assay identification of TMUV SL-retention mutants.

Figure S3. Confirmation of rescue of TMUV and JEV mutants by IFA.

Figure S4. Replicon and plaque morphology analysis of TMUV SLI- and SLIII-related mutans.

Figure S5. RNA secondary structure comparison and sequence alignment of the SLI and SLIII between CQW1 and FLSDX.

Figure S6. Effect of SD-1 3′UTR substitution on SA14 proliferation.

Figure S7. Validation of CELF2 overexpression and knockdown efficiency.

Table S1. Primers used in this study.

Table S2. Changes in the 3′UTR of TMUV after serial *in vitro* passaging.

Table S3. Proteins differentially associated with WT and ΔSLI+SLIII RNAs.

Figure S1. RNA secondary structure prediction of elements used in recombinant TMUV Domain I.

A

WT

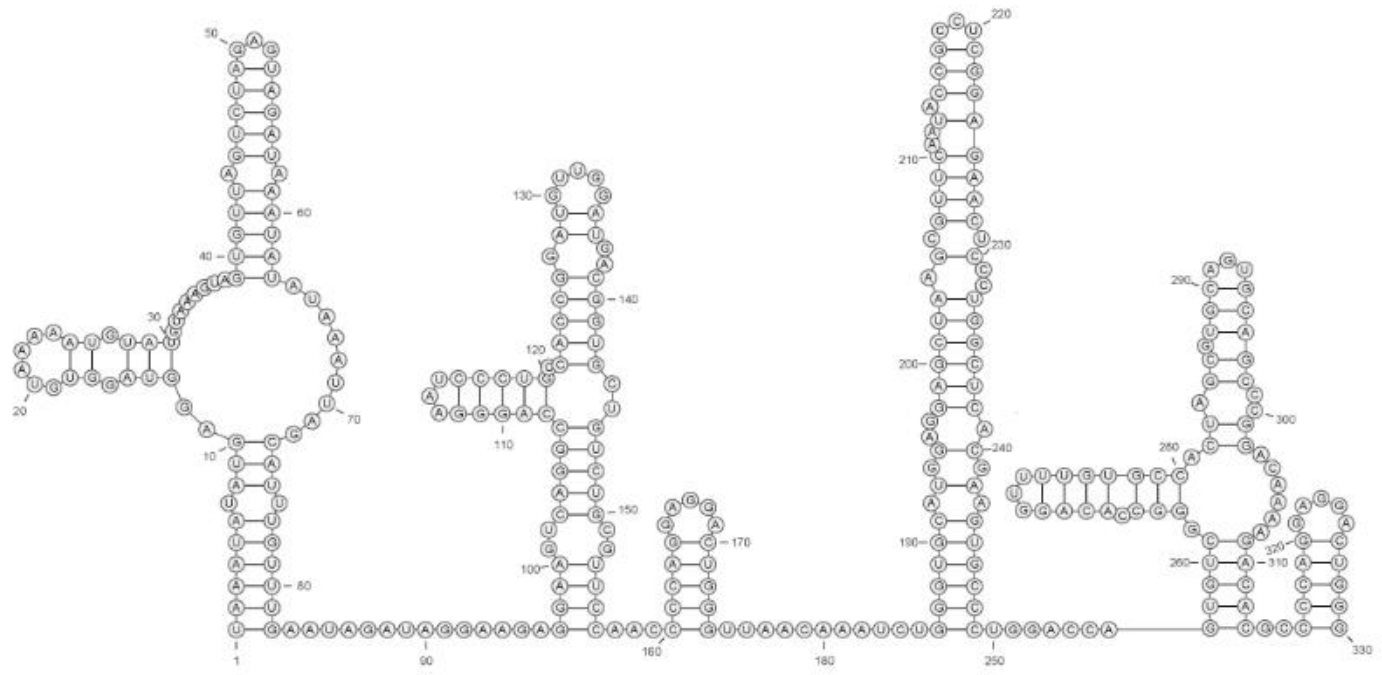

B

SLII retention

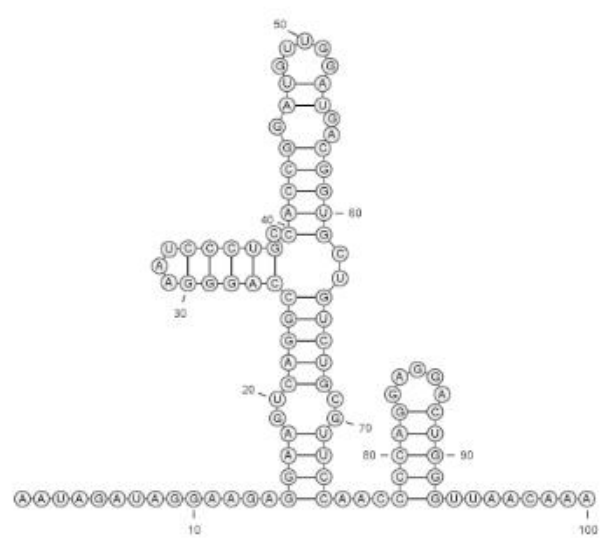

C

DENV SLI

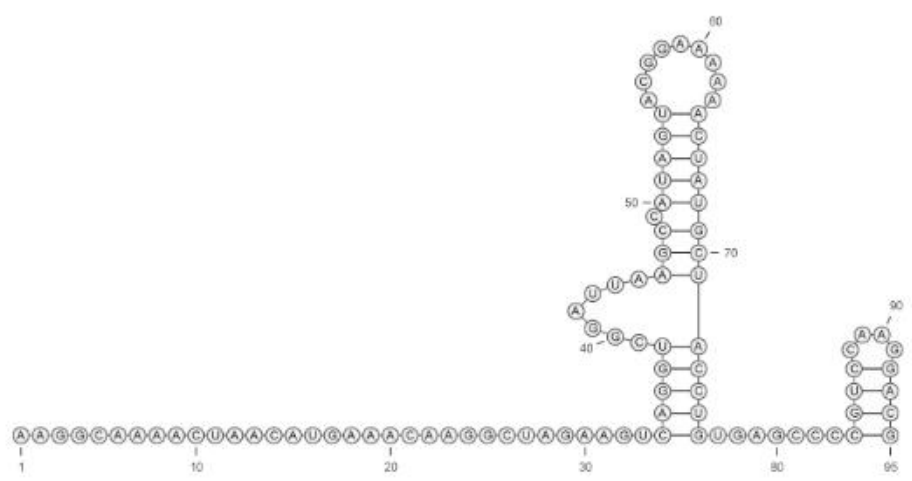

D

16S rRNA partial

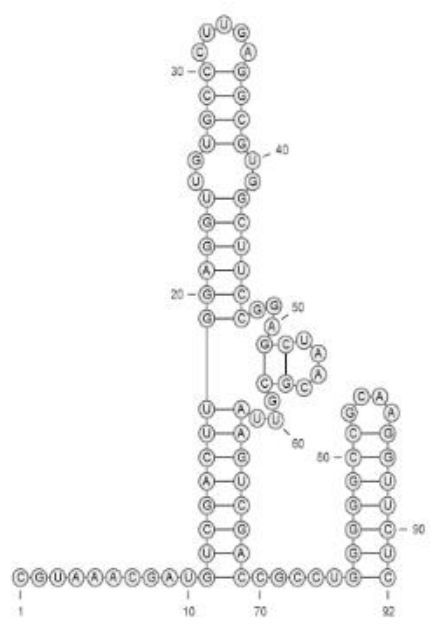

E

SLII disrupted

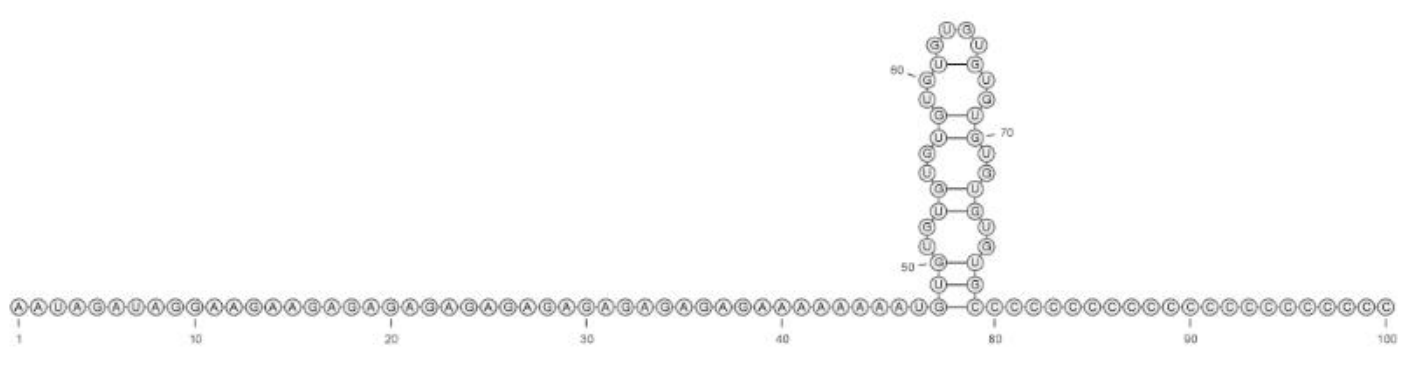

Predicted RNA secondary structures of CQW1 WT (A), SLII-retention (B), DENV SLI substitution (C), 16S rRNA partial substitution (D), and structure-disrupted SLII (E). Sequence information for CQW1 WT (GenBank ID: KM233707), DENV SLI (GenBank ID: AF038403), and 16S rRNA partial (GenBank ID: KM401419) was obtained from the NCBI database.

Figure S2. Immunofluorescence assay identification of TMUV SL-retention mutants.

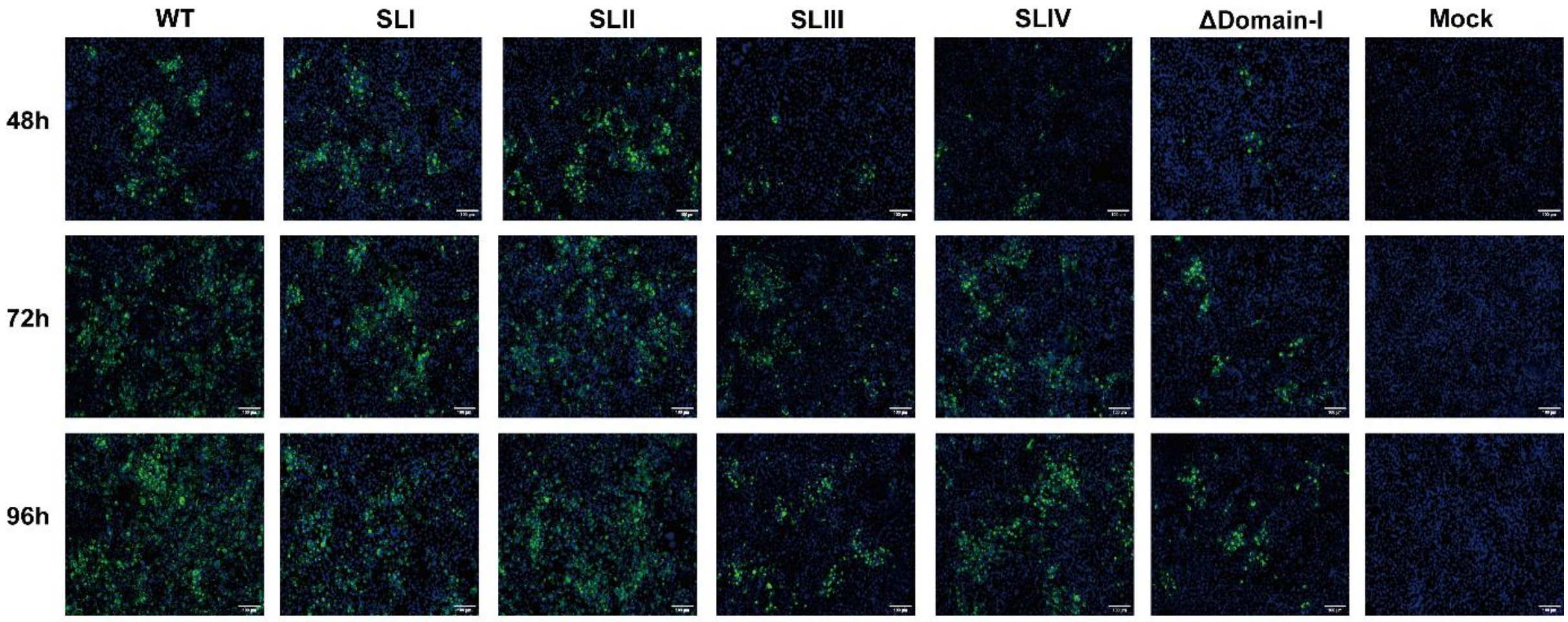

Representative immunofluorescence images showing rescue and spread of TMUV WT, its SL-retention mutants, and the mutant harboring deletion of the entire Domain I. BHK-21 cells were seeded in 12-well plates, and transfected with 0.8 μg of indicated recombinant infectious clone plasmids. At 48, 72, 96 hpt, the cells were fixed, permeabilized, and incubated with a mouse anti-TMUV (in house) as the primary antibody, followed by fluorescein isothiocyanate (FITC)-labeled goat anti-mouse IgG as the secondary antibody. Nuclei were counterstained with DAPI. Fluorescence images were acquired using a Nikon microscope. Scale bar, 100 μm.

Figure S3. Confirmation of rescue of TMUV and JEV mutants by IFA.

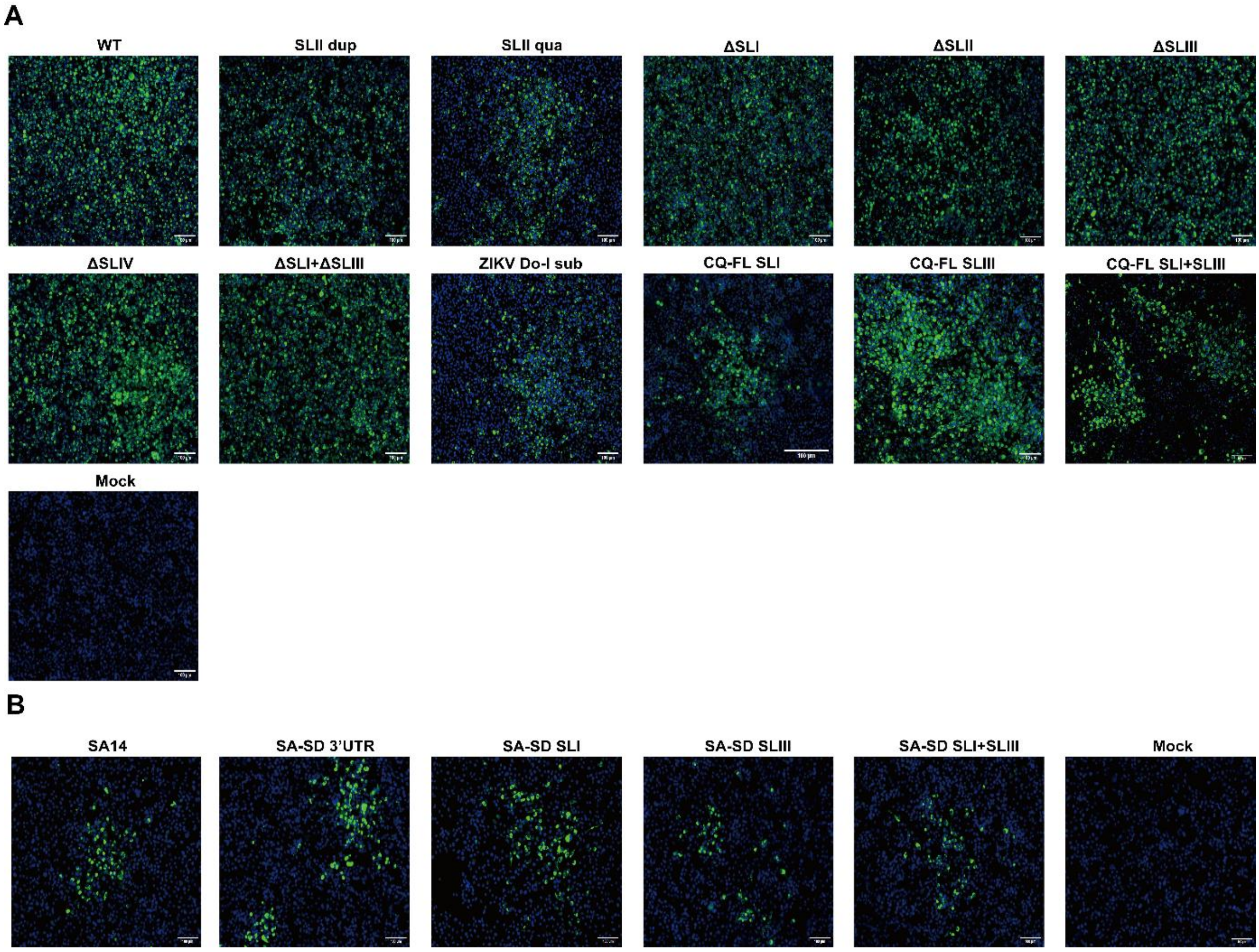

(A and B) BHK-21 cells were infected with the F0 stock of the indicated viruses. At 48 to 60 hpi, the Immunofluorescence assay was performed as described in the legend to Figure S2, except that a mouse anti-JEV polyclonal antibody (in-house) was used as the primary antibody for JEV-related mutants' identification.

Figure S4. Replicon and plaque morphology analysis of TMUV SLI- and SLIII-related mutans.

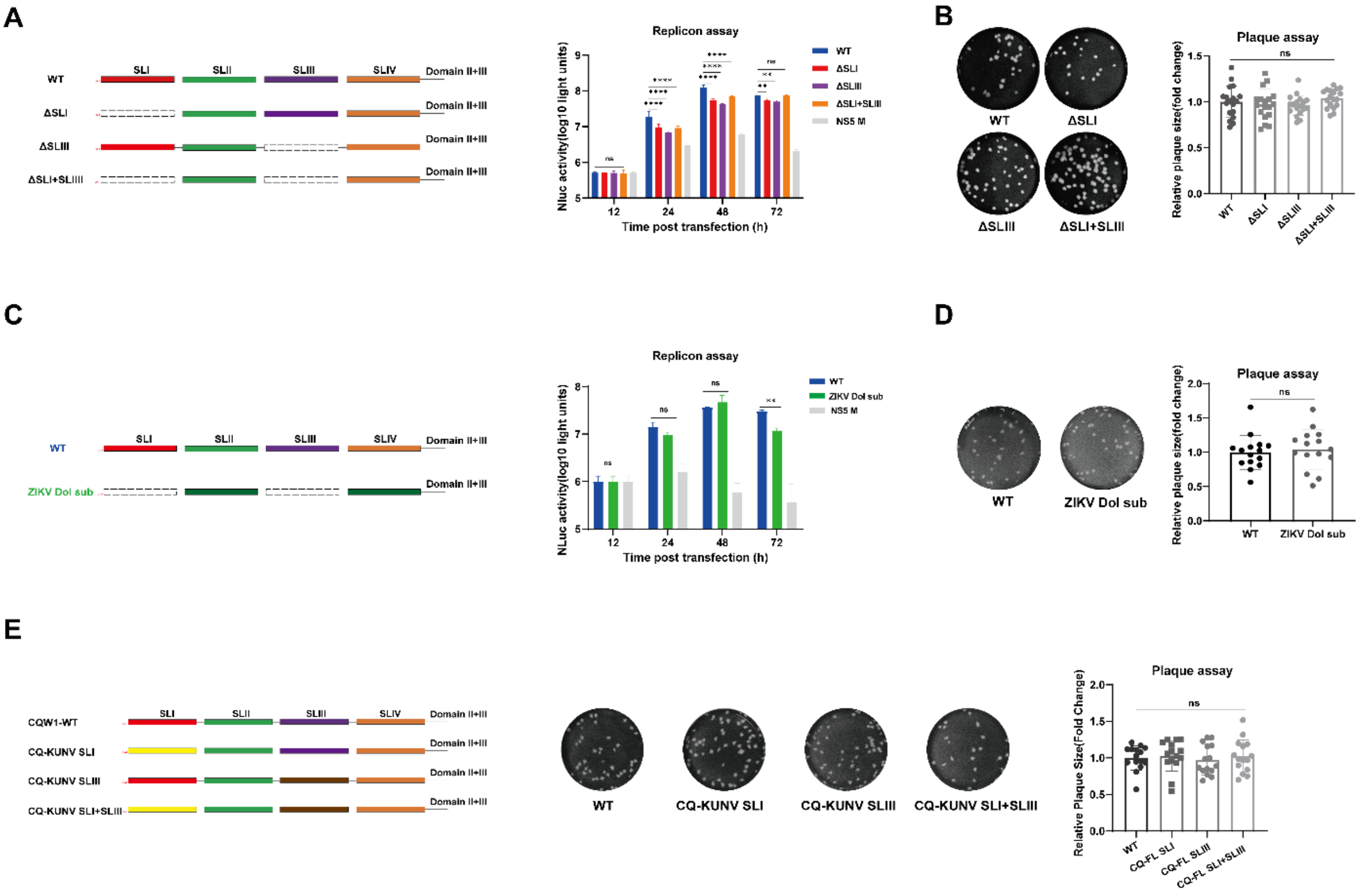

(A) Schematic representation and results of Replicon assay of WT and of SLI and/or SLIII deletion mutants. NLuc expression kinetics of WT and mutant replicons in BHK-21 cells were assessed as described in the legend to (Figure 1B-D).

(B) Plaque morphology of WT and mutant viruses in BHK-21 cells; 18 plaques per virus were randomly selected and measured using ImageJ software.

(C) Schematic representation and results of Replicon assay of WT and ZIKV Domain I substitutions. NLuc expression kinetics of WT and mutant replicons in BHK-21 cells were assessed as described in the legend to (Figure 1B-D).

(D) Plaque morphology of WT and ZIKV Domain I chimeric viruses in BHK-21 cells; 15 plaques per virus were randomly selected and measured using ImageJ software.

(E) Schematic representation and plaque morphology of WT and KUNV SLI and/or SLIII chimeric mutants in BHK-21 cells. 15 plaques per virus were randomly selected and measured using ImageJ software.

Figure S5. RNA secondary structure comparison and sequence alignment of the SLI and SLIII between CQW1 and FLSDX.

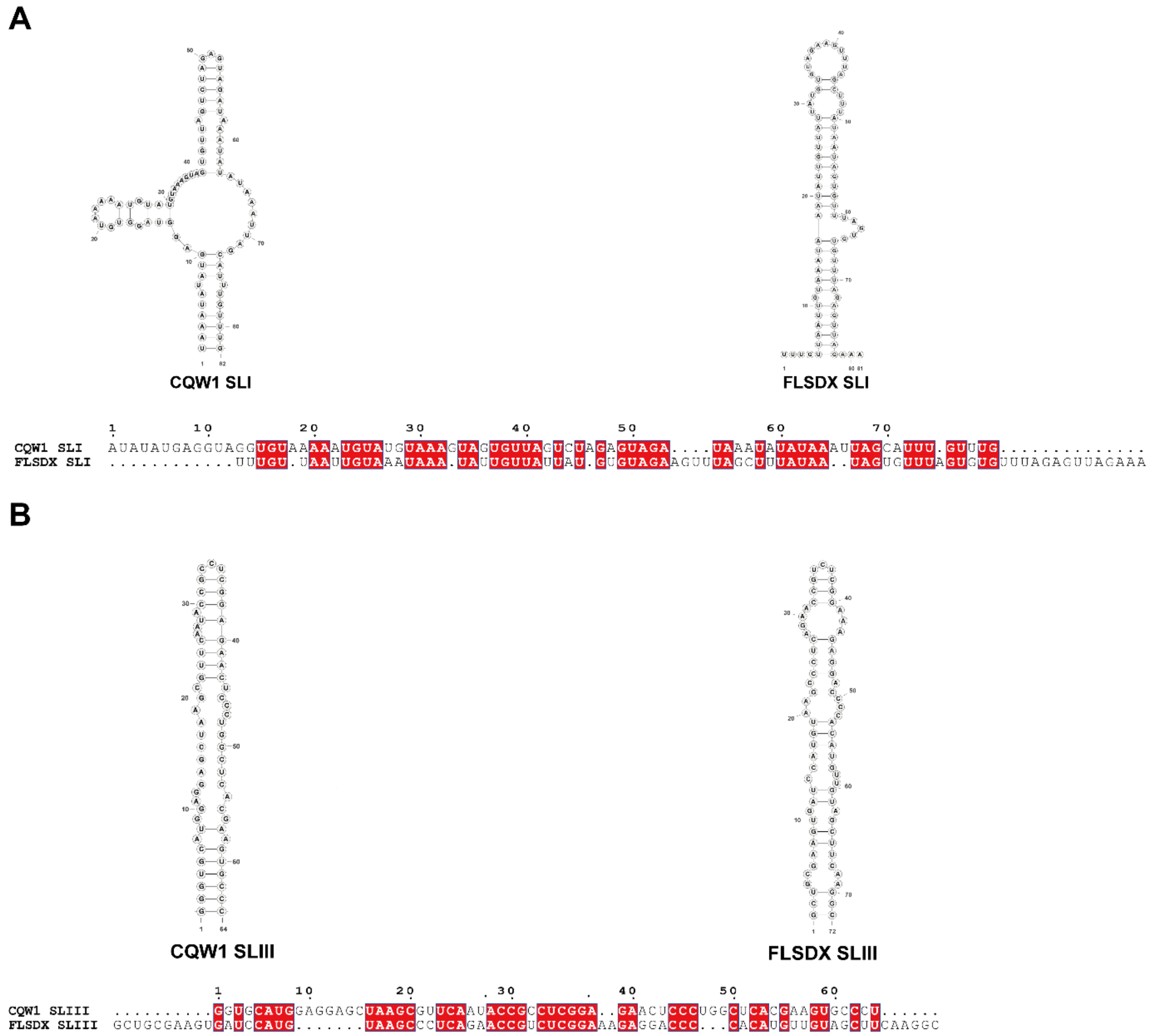

(A and B) RNA secondary structure comparison (upper) and sequence alignment (lower) of the SLI (A) and SLIII (B) between TMUV CQW1 (left) and KUNV FLSDX (right). RNA secondary structures prediction of CQW1's and FLSDX's SLI and SLIII was performed using RNAFold software, and corresponding sequence alignments were performed with ClustalW algorithms.

Figure S6. Effect of SD-1 3′UTR substitution on SA14 proliferation.

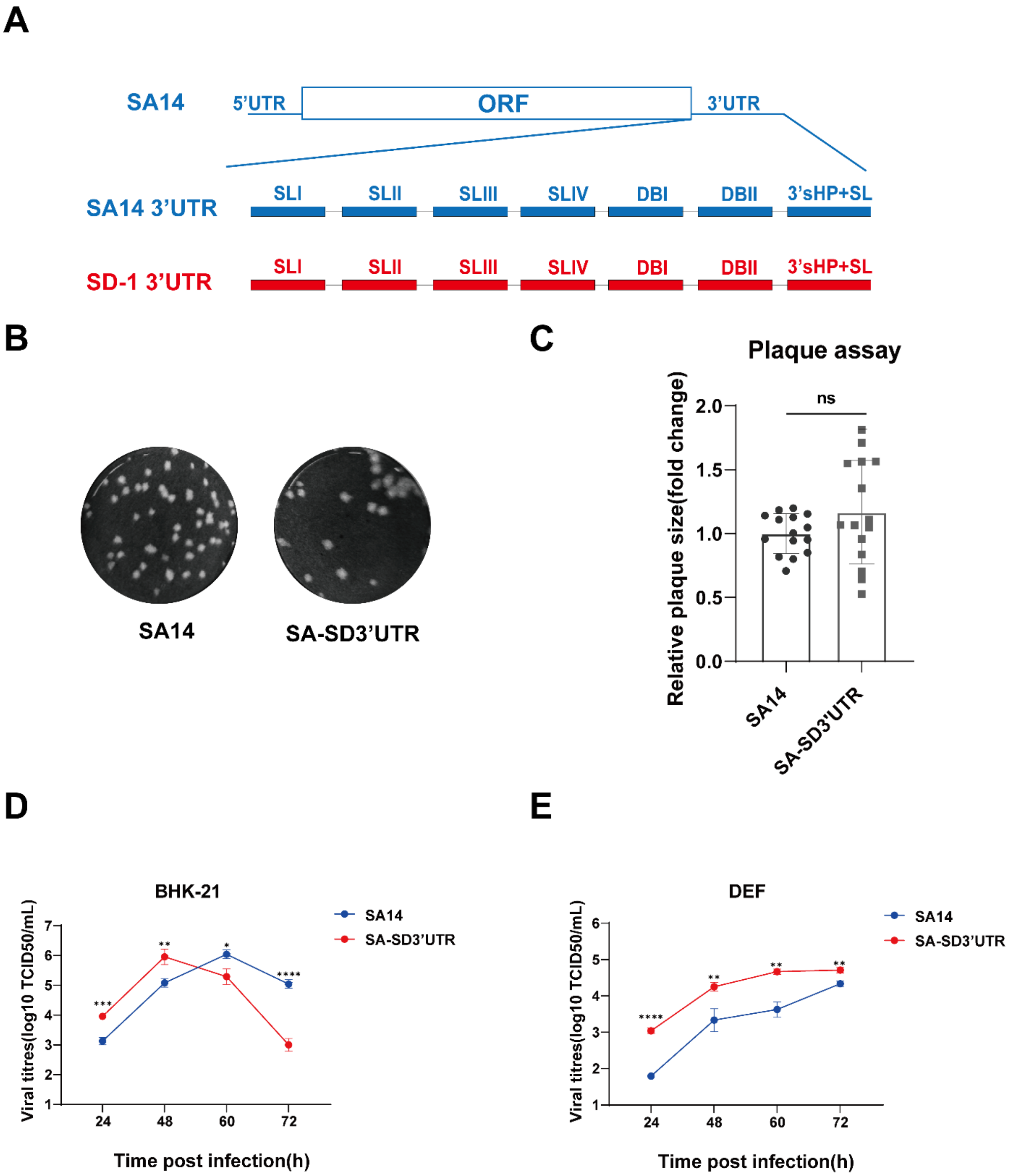

(A) Schematic diagram of SD-1 3′UTR chimeric virus generated using SA14 as the backbone.

(B and C) Plaque morphology of SA14 and SA-SD 3′ UTR viruses in BHK-21 cells, with (C) 15 plaques per virus measured using ImageJ; statistical significance was assessed by Student’s t test.

(D and E) Growth kinetics of SA14 and SA-SD 3′ UTR viruses in BHK-21 (I) and DEF (J) cells infected with 200 TCID50 of virus; statistical significance was analyzed by two-way ANOVA. Data represent the means ± SDs of three independent experiments. *, $P < 0.05$; **, $P < 0.01$; ***, $P < 0.001$; ****, $P < 0.0001$; ns, not significant.

Figure S7. Validation of CELF2 overexpression and knockdown efficiency.

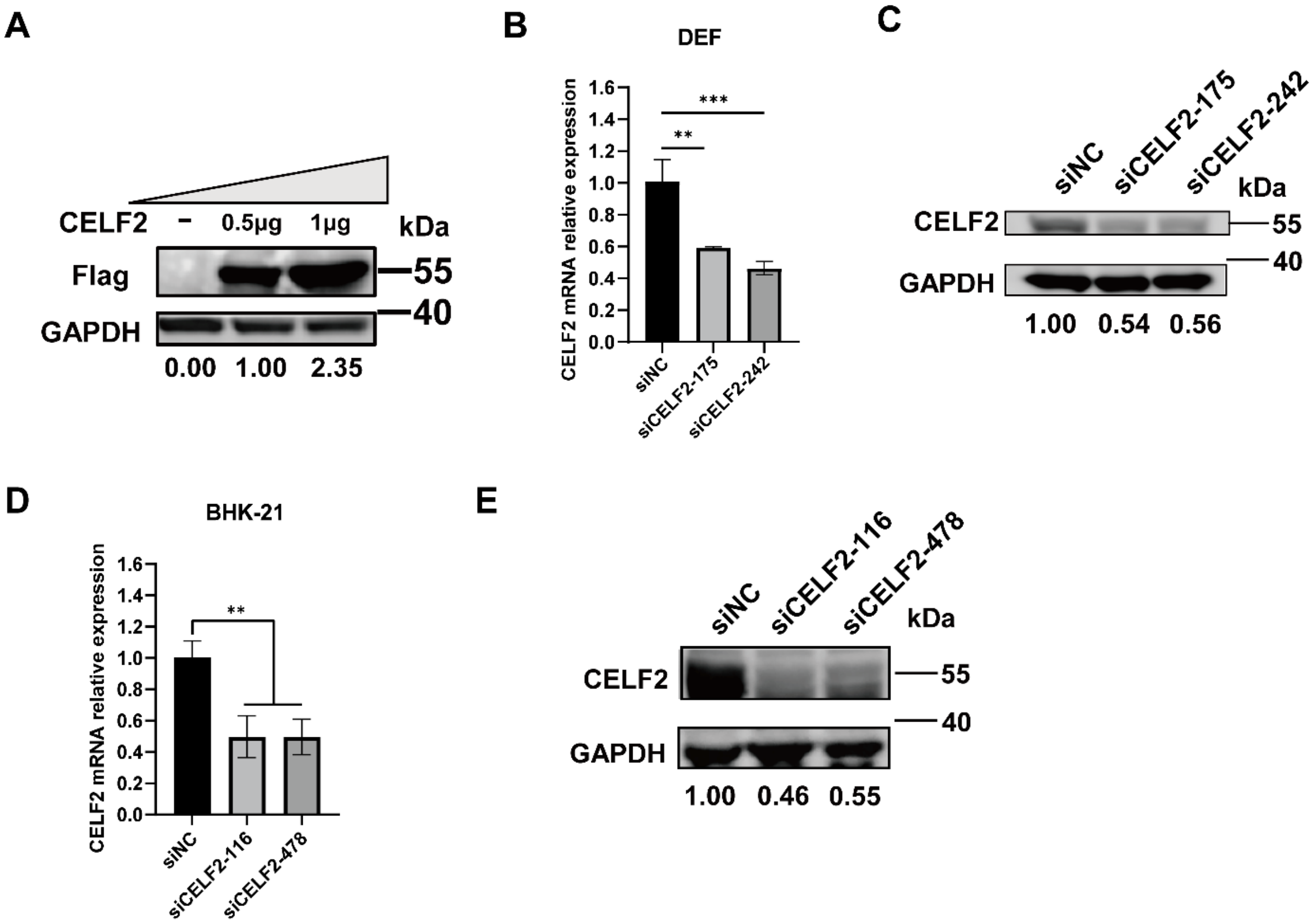

(A) DEF cells were transfected with the indicated amounts of duCELF2-Flag expression plasmid for 72 h, and the cell lysates were harvested to determine CELF2 overexpression levels.

(B and C) DEF cells were transfected with 5 pmol of duck CELF2-specific siRNAs or nonspecific control RNA for 72 h, and the knockdown efficiency of CELF2 was assessed by RT-qPCR (B) and WB (C).

(D and E) BHK-21 cells were transfected with 5 pmol of hamster CELF2-specific siRNAs or nonspecific control siRNA for 72 h, and the knockdown efficiency of CELF2 was assessed by RT-qPCR (D) and WB (E).

Table S1. Sequence of oligonucleotides used throughout the manuscript

Name	Sequence (5'-3')
CQW1-sbfI-F	CCCAGAACCACCTGCAGG
CQW1-NS5-R	TTACAAAACACCTTCACTCCAGCT
CQW1-3′UTRΔSLI-F	AGTGAAGGTGTTTTGTAAAATAGATAGGAAGAGGAAGTCAGGC
CQW1-3′UTRΔSLI~SLII-F	AGTGAAGGTGTTTTGTAATCTGGGTGCATGGAGGAG
CQW1-3′UTRΔSLI~SLIII-F	AGTGAAGGTGTTTTGTAAGGACCAGTGTCGGGCCA
CQW1-3′UTRΔSLI~SLIV-F	AGTGAAGGTGTTTTGTAAGCCGAAATGGCCCCC
CQW1-3′UTRSLI-F	TGAATAGATAGGAAGAGCCGAAATGGCCCCC
CQW1-3′UTRSLI-R	TCTTCCTATCTATTCAAACAAATGCTAA
CQW1-3′UTRSLII-F	CTGGGTTAACAAATCTGGCCGAAATGGCCCCC
CQW1-3′UTRSLII-R	CAGATTTGTTAACCCAGTCCTCCT
CQW1-3′UTRΔSLII-F	TTGAATAGATAGGAAGATCTGGGTGCATGGAGGAGC
CQW1-3′UTRΔSLII-R	TCTTCCTATCTATTCAAACAAATGCTAA
CQW1-3′UTRΔSLIII-F	TGGGTTAACAAATCTGGGACCAGTGTCGGGCCA
CQW1-3′UTRΔSLIII-R	AGATTTGTTAACCCAGTCCTCCTGGGGTTGGAACG
CQW1-3′UTRΔSLIV-F	GAAGTGCCCTGGACCAGCCGAAATGGCCCCC
CQW1-3′UTRΔSLIV-R	TGGTCCAGGGCACTTCGTG
DENV SLI Sub-R	CGTCCTTGGACGGGGCT
DENV Domain-I Sub-R	CACCTTCTCAGGTGGAGCTACAG
ZIKV Domain-I Sub-F	AGTGAAGGTGTTTTGTAAGCACCAATCTTAATGTTGTCAGG
ZIKV Domain-I Sub-R	CTCAGTGTCCTCTGAGGGGCT
CQ-FL SLI F	AGCTGGAGTGAAGGTGTTTTGTAAATACTTTGTTAATTGTAAAT
CQ-FL SLIII F	GCTGCGAAGTGATCCATGTAAG
CQ-FL SLIII R	AGGAGGACTGGGTTAACAAATCTGGCTGCGAAGTGATCC
CQW1-NruI-R	TCAACGGGAAACGTCTTGTCGCGATAAGATACATTGATGAGTT
JEV-XbaI-F	TGTGGCTTGGAGCACGGTATCTAGAGTTTGAAGCTTTGGGGTTCCT
JEV-NS5-R	CCTGTCTTCCTGGATCAAGACG
SA-SD3′UTR-F	ATCCAGGAAGACAGGGTCATCTAGGGTCATTTAGACATGATAAAGTCATGT
SA-SD SLI-F	TGGAGTCAGGCCAGCAAAAG
SA-SD SLI-R	CTTTTGCTGGCCTGACTCCA
SA-SD SLIII-F	TGCGTCTCAGTCCCAGGAG
SA-SD SLIII-R	CTCCTGGGACTGAGACGCA
JEV-SbfI-R	AACGGGAAACGTCTTGCCTGCAGGTAAGATACATTGATGAGTTTGGACAAA
pCAGGS-CELF2-F	CATCATTTTGGCAAAGCCACCATGTTTGAGCGCACTTCTGAAC
pCAGGS-CELF2-R	CTACTTATCGTCGTCATCCTTGTAATCGTAAGGTTTGCTGTCATTTTTGGA
qDuck-IFN-β-F	TCTACAGAGCCTTGCCTGCAT
qDuck-IFN-β-R	TGTCGGTGTCCAAAAGGATGT
qHuman-IFN-β-F	TTGCTCTCCTGTTGTGCTTC
qHuman-IFN-β-R	AAGCCTCCCATTCAATTGCC
TMUV qNS3-F	TAAAGAGGGAGCATACTGG
TMUV qNS3-R	GCAGGGTCTGTGAAGTGA
qDuck-β-action-F	GATCACAGCCCTGGCACC

qDuck-β-action-R	CGGATTCATCATACTCCTGCTT
qHuman-GAPDH-F	GGAGCGAGATCCCTCCAAAAT
qHuman-GAPDH-R	GGCTGTTGTCATACTTCTCATGG
qDuck-CELF2-F	GCTGACACCCAAAAGGACAAGGAG
qDuck-CELF2-R	CCGCTGAAGGCACCCAAGTTAC
qMouse-CELF2-F	TGCAGCCATGAATTCCTTGACCTC
qMouse-CELF2-R	CCGCCATACCTGAGAGCATTTGAG
qMouse-β-actin-F	AGCCATGTACGTAGCCATCC
qMouse-β-actin-R	CTCTCAGCTGTGGTGGTGAA

Table S2. Changes in the 3′UTR of TMUV after serial *in vitro* passaging.

Position	Location	Reference	Alternate	Type	Cell type
51	SLI	A	G	SNP	BHK-21
61	SLI	A	G	SNP	BHK-21
144	SLII	T	C	SNP	BHK-21
215	SLIII	C	T	SNP	BHK-21
280	SLIV	T	C	SNP	BHK-21
405	DBI	G	A	SNP	BHK-21
369	DBI	G	A	SNP	C6/36
419	DBI	A	G	SNP	C6/36
421	DBI	G	A	SNP	C6/36
435	DBII	A	T	SNP	C6/36
444	DBII	C	T	SNP	C6/36
445	DBII	A	G	SNP	C6/36
499	DBII	G	A	SNP	C6/36
505	DBII	C	CA	INS	C6/36
527	3′sHP	T	A	SNP	C6/36
543	3′SL	AC	A	DEL	C6/36

Table S3. Proteins differentially associated with WT and ΔSLI+SLIII RNAs.

Protein	Gene	Ratio	P value	Regulated Type
A0A0U2LGY1	HBD	1.715986	0.0469	up
A0A2P9JZB4	TNNI1	0.537534	0.015202	down
A0A493SS17	PLAG1	#NAME?	0	down
A0A493SST7	PSD3	0.379574	0.020268	down
A0A493ST18	BCLAF1	0.599977	0.029298	down
A0A493ST86	BTRC	3.85393	0.019377	up
A0A493STV4	CELF2	3.15774	4.78E-05	up
A0A493SUP4	ZNF638	0.612018	0.030175	down
A0A493SVH5	GLB1L	/	0	down
A0A493SXR3	THOC2	0.470852	0.001571	down
A0A493SY45		/	0	down
A0A493T039		1.508521	0.018707	up
A0A493T0E1	PABPC4	0.643332	0.046197	down
A0A493T125	ELAVL4	1.576092	0.001355	up
A0A493T2B2		1.547209	0.043132	up
A0A493T2H2	RBMS3	1.87457	0.000821	up
A0A493T316	MEX3B	3.315782	0.000133	up
A0A493T3G5	KHDRBS3	1.907964	0.001003	up
A0A493T4P9	TUT1	0.547996	0.0241	down
A0A493T501	CDC42BPB	0.473422	0.019465	down
A0A493T7F5	MCCC2	1.642691	0.006421	up
A0A493T841	TIMM13	1.541724	0.013266	up
A0A493T8K8	WDFY1	0.663419	0.010763	down
A0A493T8L8	MGST1	1.647576	0.001938	up
A0A493T8T9	PUM2	3.031955	0.010986	up
A0A493T9H7	FUBP3	3.765346	1.27E-06	up
A0A493T9X1	HNRNPD	2.755207	3.45E-05	up
A0A493TA33		2.569987	0.000264	up
A0A493TAE0	RBM39	0.559542	0.004391	down
A0A493TBE9	LEMD3	1.56827	0.04279	up
A0A493TCF3		1.777649	0.021902	up
A0A493TD77	FBXW11	2.605203	0.011862	up
A0A493TEW0	SLTM	0.618304	0.018051	down
A0A493TF93		0.341102	0.040987	down
A0A493TGN8	LPP	1.527712	0.025054	up
A0A493TH04	COX5A	1.656682	0.01122	up
A0A493TKT5	KHDC4	1.803793	0.028429	up
A0A493TLY3	RBMS1	1.867041	0.004589	up
A0A493TPL3	THOC1	0.558137	0.028081	down
A0A493TRE2	GNG7	1.681835	0.021169	up
A0A493TRJ0	TIA1	1.645495	0.001251	up

A0A493TU65	PRPF4B	0.255914	0.001485	down
A0A493TUM2	BNIP1	0.529569	0.025208	down
A0A493TY91		2.251596	0.022434	up
A0A7K7KJQ3	Fam234a_0	2.102697	0.036974	up
A0A7K7KP59	Uqcrh	1.604619	0.01557	up
A0A7K7KSG4	Prpf8	0.31596	0.027184	down
A0A7K7KSI3	Snu13	0.646387	0.000178	down
A0A7K7KSK5	Snrpd1	2.439312	0.013318	up
A0A7K7KU25	Snrpb2	2.211469	0.001967	up
A0A7K7KV62	Gem	/	0	down
A0A7K7KV87	Uqcrb	1.69213	0.013174	up
A0A7K7KVE6	Hpcal1	1.544535	0.009474	up
A0A7K7KZH2	Pum1	2.042475	6.70E-05	up
A0A7K7L1G9	Snrpd3	2.498045	0.008575	up
A0A7K7L3Z9	Eftud2	0.378514	0.020376	down
A0A7K7L418	Thoc7	0.108808	0.000636	down
A0A7K7L450	Wdr18	0.564269	0.000735	down
A0A7K7L6A6	Celf1	3.671813	0.004941	up
A0A7K7L700	Nars	9.031387	0.001912	up
A0A7K7L7A4	Prpf6	0.266249	0.013095	down
A0A7K7L7D0	Acly	0.426807	0.037805	down
A0A7K7L7X2	Snrpg	2.563874	4.84E-05	up
A0A7K7L8X1	Snrpb	2.335166	0.000314	up
A0A7K7LEE3	Sec31a	0.229562	0.021197	down
A0A7K7LEG5	Ankrd17	0.510716	0.003559	down
A0A7K7LH36	Huwe1_1	0.361125	0.042712	down
A0A7K7LHR6	Cox6b1	1.626317	0.011905	up
A0A7K7LJE1	Pola2_0	0.643905	0.034972	down
A0A7K7LKL6	Brix1	0.634338	0.021016	down
A0A7K7LLS3	Pid1	0.465477	0.019967	down
A0A7K7LM00	Tex10	0.561505	0.047229	down
A0A7K7LNG6	Tnni2	0.565441	0.010802	down
A0A7K7LNW2	Pccb	1.746766	0.005088	up
A0A8B9R4D3	ZFP36L2	1.864369	0.047287	up
A0A8B9R536	HNRNPDL	2.88723	4.81E-06	up
A0A8B9R5Z2		0.239842	0.005428	down
A0A8B9R612		1.567201	0.044471	up
A0A8B9R725	BCAP29	0.380403	0.018992	down
A0A8B9SJE4	MRPS36	1.724088	0.014808	up
A0A8B9SJZ4		1.550978	0.00033	up
A0A8B9SLP8		1.784061	0.040187	up
A0A8B9SNJ8		1.602465	0.01188	up
A0A8B9STJ1		1.54497	0.000178	up

A0A8B9SUX1	ZNF207	1.86154	0.001208	up
A0A8B9SW66		2.376357	0.001956	up
A0A8B9SWU5		0.598114	0.009849	down
A0A8B9SXD1		0.320701	0.000276	down
A0A8B9T025	FUBP3	3.512697	1.53E-07	up
A0A8B9T0V6		1.927529	0.003233	up
A0A8B9T4S1	UNG	1.510688	0.007702	up
A0A8B9TC78	LUC7L3	1.798505	0.002866	up
A0A8B9TCG3	PURA	0.563466	0.036996	down
A0A8B9TEU9		1.807279	0.011917	up
A0A8B9TH83		0.396843	0.001029	down
A0A8B9TKD4		0.496194	0.032888	down
A0A8B9TMZ8	SNRPC	4.492456	0.001318	up
A0A8B9TPG8	LSM8	0.531041	0.01643	down
A0A8B9TSA6		1.753253	0.002059	up
A0A8B9TVC1		2.708729	0.027534	up
A0A8B9TVW9		1.57792	0.004955	up
A0A8B9U2M5		0.22687	6.34E-05	down
A0A8B9UDA5		0.338633	0.008806	down
A0A8B9UMQ4		0.229632	0.003855	down
A0A8B9UMZ3		0.593869	0.000245	down
A0A8B9UWS6		1.510118	0.031822	up
A0A8B9V2D7		0.390843	0.026313	down
A0A8B9VMZ9		0.459708	0.002338	down
A0A8B9VQ41		3.863181	0.003424	up
A0A8B9VWI8		2.881587	0.001481	up
A0A8B9ZHZ0	FUBP1	1.910148	0.000173	up
A0A8B9ZT73		7.234918	5.61E-06	up
A0A8B9ZUJ0		0.615973	0.014672	down
A0A8B9ZYK9		0.293765	0.009065	down
A0A8C3B8U1		0.549006	0.012007	down
A0A8C3BGE5		0.420904	0.000317	down
A0A8C3BHA0		4.149072	0.000498	up
A0A8C3BIC7		0.179667	0.0076	down
A0A8C3BJA3		0.665879	0.019374	down
A0A8C3C3F1		4.287683	3.49E-06	up
A0A8C3C7V4	SNRPC	3.461454	0.000162	up
A0A8C3C7W4		2.51288	5.47E-06	up
A0A8C3C855		1.566983	0.005001	up
A0A8C3CUI7		1.500659	0.003217	up
A0A8C3GKF4		0.32544	0.003041	down
A0A8C3GMP6		1.513139	0.000154	up
R0JTY6	Anapl_02473	1.703382	0.035404	up

R0KJM7	Anapl_18874	1.623415	0.015776	up
R0KXT3	Anapl_16928	0.363207	0.021718	down
R0LKH1	Anapl_01213	4.058045	0.004042	up
U3IGT3	GEMIN5	1.51412	0.035727	up
U3IMN8	PITPNB	1.721247	0.033668	up
U3IP43	P4HA1	0.515025	0.03727	down
U3IQK3	SNRNP40	0.32571	9.24E-05	down
